# Chaotic Mountain Gazelle Optimizer Improved by Multiple Oppositional-Based Learning Variants for Theoretical Thermal Design Optimization of Heat Exchangers Using Nanofluids

**DOI:** 10.3390/biomimetics10070454

**Published:** 2025-07-10

**Authors:** Oguz Emrah Turgut, Mustafa Asker, Hayrullah Bilgeran Yesiloz, Hadi Genceli, Mohammad AL-Rawi

**Affiliations:** 1 Department of Industrial Engineering, Faculty of Engineering and Architecture, Izmir Bakircay University, Menemen, İzmir 35665, Türkiye; oguzemrah.turgut@bakircay.edu.tr; 2 Department of Mechanical Engineering, Faculty of Engineering, Aydın Adnan Menderes University, Efeler, Aydın 09010, Türkiye; 3 Graduate School of Natural and Applied Sciences, Aydın Adnan Menderes University, Efeler, Aydın 09010, Türkiye; 4 Faculty of Mechanical Engineering, Yıldız Technical University, Istanbul 34349, Türkiye; hgenceli@yildiz.edu.tr; 5 School of Computing, Mathematics & Engineering, Charles Sturt University, Bathurst, NSW 2795, Australia

**Keywords:** Mountain Gazelle Optimizer, nanofluids, opposition-based learning, chaotic systems, shell and tube heat exchangers

## Abstract

This theoretical research study proposes a novel hybrid algorithm that integrates an improved quasi-dynamical oppositional learning mutation scheme into the Mountain Gazelle Optimization method, augmented with chaotic sequences, for the thermal and economical design of a shell-and-tube heat exchanger operating with nanofluids. The Mountain Gazelle Optimizer is a recently developed metaheuristic algorithm that simulates the foraging behaviors of Mountain Gazelles. However, it suffers from premature convergence due to an imbalance between its exploration and exploitation mechanisms. A two-step improvement procedure is implemented to enhance the overall search efficiency of the original algorithm. The first step concerns substituting uniformly random numbers with chaotic numbers to refine the solution quality to better standards. The second step is to develop a novel manipulation equation that integrates different variants of quasi-dynamic oppositional learning search schemes, guided by a novel intelligently devised adaptive switch mechanism. The efficiency of the proposed algorithm is evaluated using the challenging benchmark functions from various CEC competitions. Finally, the thermo-economic design of a shell-and-tube heat exchanger operated with different nanoparticles is solved by the proposed improved metaheuristic algorithm to obtain the optimal design configuration. The predictive results indicate that using water + SiO_2_ instead of ordinary water as the refrigerant on the tube side of the heat exchanger reduces the total cost by 16.3%, offering the most cost-effective design among the configurations compared. These findings align with the demonstration of how biologically inspired metaheuristic algorithms can be successfully applied to engineering design.

## 1. Introduction

There has been an ongoing and relentless pursuit of finding efficient and adaptive problem-solving strategies in engineering design problems. Although many valuable efforts have been made to overcome this challenging issue, nature remains the leading option and continues to inspire research in this area. Biomimetics is an exemplary interdisciplinary field in this realm, simulating models, systems, and elements of nature to solve complex problems and has significantly contributed to the development of state-of-the-art algorithms, materials, and architectural design [1,2]. Biomimetics is based on the fundamental scientific principle that the evolution of individuals over millions of years has fine-tuned biological systems to perform advanced tasks under severe constraints. This accumulated evolutionary knowledge is utilized as an innovative guide for designing artificial systems that can exhibit similar adaptability and efficiency [3].

Biological inspiration from different natural sources has led to the rise of metaheuristic algorithms, a class of high-level, nature-inspired, problem-independent solvers capable of navigating highly complex, multidimensional search spaces. Metaheuristic algorithms are inspired by natural processes and derive their primary structures from the biological evolution [4], food search [5], and social behaviors [6] of animals, as well as biochemical interactions [7]. There is a profound and reciprocal synergy between biomimetics and metaheuristics, promoting the development of each scientific topic in a mutually supportive way. The components of biomimetics provide a conceptual framework for developing intelligently devised metaheuristic algorithms that can mimic sophisticated biological phenomena such as swarm intelligence, tool use, or camouflage. On the other hand, metaheuristic algorithms serve as a complementary tool in terms of optimization purposes, such that they are capable of solving challenging problems in biomimetic-based design, such as the aerodynamics of UAV design inspired by the flapping of bird wings [8], the self-cleaning of wetted surfaces through lotus leaves [9], or the thermoregulation of systems via terms of termite mounds [10]. This mutual collaboration has led to the emergence of a novel research area, focusing on the development of nature-inspired new generation algorithms that not only simulate the activities of living creatures but also benefit from the intrinsic problem-solving strategies encountered in daily life.

Metaheuristic algorithms are traditionally classified into two subgroups: modern and classical algorithms. According to this classification, algorithms developed before the 1990s are considered classical, while those created afterward are referred to as modern optimization methods. Current metaheuristic algorithms generally rely on the source of inspiration from which they originated. They can be categorized into four main sub-branches: Evolutionary Algorithms [4], Swarm Optimization Algorithms [6], Physics-Based Algorithms [11], and Human-Based Algorithms [12]. Each algorithm available in the literature can be used to solve a diverse range of real-world optimization problems, each with its advantages and disadvantages. The No Free Lunch (NFL) theorem [13] posits that no specific optimization algorithm can address all optimization problems. Therefore, researchers focus on finding alternative methods to overcome the challenges posed by NFL theory, which necessitates the development of a robust optimization framework capable of yielding promising results for the defined optimization problem. One alternative to enhance the search capacity of any algorithm is to hybridize it with another metaheuristic that possesses complementary characteristics to offset the deficiencies of the base algorithm [14]. Integrating chaos into the original metaheuristic optimizer by replacing uniformly distributed random numbers with sequential chaotic numbers is another method to improve the search effectiveness of the algorithm. The straightforward implementation of chaotic numbers in any metaheuristic algorithm significantly broadens their applicability, supported by their non-repetitive and ergodic numerical properties, which can enable the algorithm to perform iterative random searches at greater speeds [15]. The basic principles of Opposition-Based Learning concepts and their various algorithmic variants have been effectively incorporated into different metaheuristic algorithms to date, thanks to the efficiency of the solutions generated by using opposite numbers, which encourages the algorithm to identify candidate solutions very close to the global optimum point [16].

One of the main concerns of this research study is to examine the enhanced thermo-economic performance optimization of shell and tube heat exchangers using various types of nanofluids through the proposed novel hybrid optimizer. The literature is gradually improving with an increasing number of experimental and theoretical studies on nanofluid-integrated refrigerants that drive the heat transfer mechanism of shell-and-tube heat exchangers. Researchers have explored the potential applications of various types of nanofluids in operating streams. They utilized single- or multi-objective design optimization to evaluate the configurations and identify the most suitable design parameters, including the volumetric ratio of suspended nanoparticles [17,18,19]. This research study proposes an alternative solution strategy to address the rigid assumptions of the NFL theorem by concurrently integrating a novel mutation scheme based on different variants of Opposition-Based Learning and pseudorandom-chaotic numbers generated from various chaotic maps into the recently developed metaheuristic, the Mountain Gazelle Optimizer (MGO). This is a new optimization method developed by Abdollahzadeh et al. [20] that simulates the social life of mountain gazelles and establishes a characteristic hierarchy among them. Despite its relatively recent development, this algorithm has been applied to several engineering design problems [21,22,23]. Although this optimizer has many algorithmic advantages, such as balanced search strategies, rapid convergence to optima in early iterations due to aggressive exploration, and low parameter sensitivity, the specific shortcomings of this algorithm must be meticulously addressed, including the lack of an adaptive search mechanism to guide the transition between exploration and exploitation, leading to premature convergence at local points in high-dimensional optimization problems. To alleviate the algorithmic drawbacks of this algorithm, an intelligently devised hybrid method is proposed and integrated into the standard Mountain Gazelle Optimizer. The first step in hybridization involves a comprehensive population initialization strategy that integrates Latin Hypercube Sampling [24] with chaotic number-based randomization and the principles of Opposition-Based Learning [25] to generate trial candidate solutions. The second step in algorithm development focuses on evaluating the optimization performance of various chaotic variants of the MGO algorithm to identify the best-performing chaotic method among the twenty-one chaos-induced MGO optimizers. Forty-eight artificially generated multidimensional optimization benchmark problems, comprising twenty-four unimodal and multimodal test cases, have been utilized to determine which chaotic MGO optimizer among the competing chaotic algorithm alternatives produces the most accurate predictions. The third procedural step of algorithm development primarily involves creating a novel mutation scheme, based on the valuable contributions of two variants of the Quasi-Dynamic Opposition-Based algorithm [26], guided by an intelligently designed adaptive switch mechanism. Chaotic numbers generated from the best-performing chaotic map and the proposed novel mutation scheme have been integrated into the original MGO algorithm to enhance its overall optimization capability regarding solution accuracy and robustness. Complex benchmark instances taken from the suite of CEC 2013 and CEC 2014 test problems with varying dimensionalities have been solved using the proposed method, which will then be evaluated on artificially generated test problems used in the CEC 2006 competitions. Finally, a real-world benchmark case related to the thermo-economic design of a shell-and-tube heat exchanger operating with different nanofluids will be simulated, and optimal decision parameters that minimize the total cost of the heat exchanger while satisfying operational constraints will be determined. This case involves numerous restrictive design constraints and includes integer and continuous decision parameters that must be meticulously optimized. This research study aims to introduce four significant novelties to the existing literature, which the following terms can briefly convey.

- Proposing a novel framework for initial population generation that integrates chaotic Latin Hypercube Sampling with the foundational principles of Opposition-Based Learning.
- Evaluating the optimization efficiency of twenty-one different chaotic Mountain Gazelle Algorithms and determining which chaotic method produces the most accurate predictions.
- Developing an innovative dexterous mutation scheme utilizing two efficient variants of an Opposition-Based Learning search mechanism, coordinated by an adaptive switch mechanism, and incorporating this manipulative search equation into the Chaos-Assisted Mountain Gazelle Optimizer.
- Maintaining the thermal design of shell and tube heat exchangers involves working with various nanoparticles in the tube bundle through the proposed enhanced Mountain Gazelle Optimizer.

The remaining sections of this research study are organized as follows: Section 2 explains the fundamentals of the Mountain Gazelle Optimizer. Section 3 introduces the preliminaries of the chaotic algorithms and the procedural integration steps of various chaotic maps into the original MGO, explaining the numerical experiments used to determine the best chaotic algorithm among competing alternatives. Section 4 outlines the fundamental algorithmic steps of the proposed mutation scheme and describes its integration into the chaos-induced MGO. Section 5 evaluates the optimization performance of the improved algorithm using various multidimensional benchmark problems. Section 6 focuses on determining the most suitable topological design parameters for shell-and-tube heat exchangers operating with different nanoparticles. This topic has not been studied in-depth in past literature approaches. Section 7 concludes this study with notable comments and outlines future directions for upcoming studies on the thermal design of nanofluid-based heat exchangers and the development of OBL-based mutation schemes.

## 2. Fundamentals of Mountain Gazelle Optimizer

The social herding behaviors of mountain gazelles inspire the development of the Mountain Gazelle Optimization algorithm. The basic concepts of their inhabitation in the natural habitat are the main elements behind the algorithm’s development and the production of the governing mathematical model. MGO algorithm performs four distinctive operators during trial solution generation, considering the influential factors of bachelor male herds, maternity herds, solitary and territorial males, and migrating mountain gazelles while searching for available food sources. Each trial member of the population (*X_i_*) can be produced among the subgroups of maternity herds, bachelor male herds, or solitary males during the iterative process. The adult male gazelle generated from the alternative subgroup of herds is considered the best solution for the current iteration. The algorithm identifies one-third of the entire population as part of the subpopulation of young gazelles, which also has the highest fitness cost. Other solutions extracted from the evolving gazelle population are considered to belong to the subgroup of maternity herds. The fittest (strongest) gazelles with high-quality solutions are preserved to be considered for the upcoming generations. The remaining lower-cost solutions are considered sick and old gazelles and are selected from the iteratively adjusted gazelle population. The algorithm executes the exploration and exploitation phases in parallel, two of which are run by the four main search mechanisms, which also makes it possible for the search agents to probe around the so-far-obtained best solution to perform exploitation as well as create high and unexpected jumps to avoid local pitfalls to maintain a reliable exploration along the search space. The subsections below explain the details of the responsible search mechanisms associated with different categories of herding gazelles.

### 2.1. Territorial Solitary Males

When newborn gazelles reach adulthood and become strong, they establish their territory and mark spatial distances to separate themselves from neighboring gazelles. A fierce battle erupts between these solid and young gazelles over the occupation of the territory or possession of the females. Young gazelles strive to capture the territory currently occupied by the established individuals, while adult males attempt to defend their territories. The defined search equation mathematically models this dispute between adult and young gazelles

(1) $$TSM={Male}_{G}-\left|\left({rdint}_{1}\cdot MCV-{rdint}_{2}\cdot X^{iter}\right)⊙FF\right|⊙{Cof}_{1,r}$$

In the above equation, *Male_G_* is the best solution obtained throughout the iterative process; model parameters *rdint*_1_ and *rdint*_2_ are randomly chosen integers between 1 and 2; *MCV* is an algorithm parameter called young male herd coefficient, calculated by Equation (2); *FF* is another model coefficient computed using Equation (3); *Cof*_1,*r*_ is randomly generated coefficient vector renewed in each iteration to enhance the search efficiency of the algorithm, calculated by Equation (4); the symbol ⊙ represents a dot product that performs the multiplication of D-dimensional vectors; and the term $\left|\left({rdint}_{1}\cdot MCV-{rdint}_{2}\cdot X^{iter}\right)⊙FF\right|$ is the absolute value operator.

(2) $$MCV=X_{rs}\cdot {rd01}_{1}+M_{pr}\cdot {rd01}_{2},\;\;\;\;rs=\left\{\left\lceil \frac{N}{3}\right\rceil ,\ldots ,N\right\}$$

In Equation (2), *N* is the number of gazelle in the population; *X_rs_* is a random solution selected from young males within the interval of *rs*; *M_pr_* is the mean value of search agents, which are randomly chosen from the interval between *N*/3 and *N*; random parameters *rd*01_1_ and *rd*01_2_ are randomly chosen integers between 0 and 1; and $\left\lceil x\right\rceil$ is the ceiling function that maps the real number “x” to the smallest integer greater than or equal to that number.

(3) $$FF=S(D)\cdot exp\left(2-iter\cdot \left(\frac{2}{Maxiter}\right)\right)$$

where *S*(*D*) is a set of *D*-dimensional random numbers generated from the uniform distribution; *exp*() is the mathematical exponential function; and *Maxiter* is the maximum number of iterations, while *iter* is the current iteration counter.

(4) $${Cof}_{r}=\left\{\begin{array}{c} \left(aa+1\right)+{rnd}_{1}\\ \begin{array}{c} aa\cdot S_{2}\left(D\right)\\ \begin{array}{c} {rnd}_{3}\left(D\right)\\ S_{3}\left(D\right)⊙{S_{4}\left(D\right)}^{2}⊙cos\left(2\cdot {rnd}_{2}\cdot S_{3}\left(D\right)\right) \end{array} \end{array} \end{array}\right.$$

In Equation (4), *aa* is an iteratively adjusted parameter calculated by Equation (5); *rnd*_1_ and *rnd*_2_ are random numbers within the range 0 and 1; and *rnd*_3_(*D*) is a *D*-dimensional random number from a uniform distribution.

(5) $$aa=-1+iter\cdot \left(\frac{-1}{Maxiter}\right)$$

### 2.2. Maternity Herds

Maternal gazelles play a crucial role in the sustainable life cycle of mountain gazelles, as they give birth to the young males that form the herding population. Male gazelles also influence the rearing of newborn male gazelle offspring and the behavior of female gazelles. The following expression can translate this gazelle behavior into a mathematical equation.

(6) $$MH=\left(MCV+{Cof}_{2,r}\right)+\left({rdint}_{3}\cdot {Male}_{G}-{rdint}_{4}\cdot X_{rand,N}\right)⊙{Cof}_{3,r}$$

In Equation (6), *MCV* contributes to the influence of young males into the gazelle population, which was previously explained in Equation (2); *Cof*_2,*r*_ and *Cof*_3,*r*_ are coefficient vectors independently calculated by using Equation (4); parameters *rdint*_3_ and *rdint*_4_ are random integers between 1 and 2. *Male_G_* is the gazelle with the best fitness value for the current iteration, and *X_rand_*_,*N*_ is the random gazelle chosen from the gazelle population.

### 2.3. Bachelor Male Herds

The gradual maturation of young male gazelles leads to them owning a specific territory and possessing female gazelles. Young gazelles engage in a fierce battle with the male gazelles over control and possession of the females during this algorithm phase, as formulated by the following.

(7) $$BMH=\left(X^{iter}-DD\right)+\left({rdint}_{5}\cdot {Male}_{G}-{rdint}_{6}\cdot MCV\right)⊙{Cof}_{4,r}$$

In Equation (7), *X^iter^* is the current position of the gazelles for the current iteration *iter*; *Cof*_4,*r*_ is a randomly generated coefficient vector independently calculated by using Equation (4); *rdint*_5_ and *rdint*_6_ are integers randomly chosen from 1 and 2; and *DD* is calculated by

(8) $$DD=\left(\left|X^{iter}\right|+\left|{Male}_{G}\right|\right)\cdot \left(2\cdot {rnd}_{4}-1\right)$$

where *rnd*_4_ is a random number within the range [0, 1].

### 2.4. Migration for Searching Food

Mountain gazelles in the population look for fertile areas where food sources are likely to be abundant. They travel along the possible paths to reach these promising areas. Randomly generated solutions between upper and lower search limits are utilized to formulate this foraging behavior, as shown in the following.

(9) $$MSF=LB+{rnd}_{5}\cdot \left(UB-LB\right)$$

where *rnd*_5_ is a random value between 0 and 1, and *LB* and *UB* are the lower and upper limits of the defined search space. These four defined search mechanisms are applied to all gazelle population members to generate new solutions for offspring. After producing new gazelle individuals, each generation’s solutions are reordered in ascending order. The best gazelles, having the fittest solution values, remain in the population, while the gazelle members with the worst fitness values are removed from the population. The best gazelle can also be considered the adult male that owns the territory in terms of dominance.

## 3. Chaotic Mountain Gazelle Optimization Algorithm

Integrating chaos into metaheuristic algorithms has been a widespread application for nearly two decades, augmenting the efficiency of the base algorithm by maintaining a proper balance between exploitation and exploration search mechanisms, two essential features of any metaheuristic optimizer. In general, chaos can be defined as an unpredictable yet practical approach to influencing the sequential behaviors of any natural event. Minimal changes in the initial conditions may result in a considerable deviation in the nonlinear characteristics of the system’s future behavior. Chaotic systems can also be mathematically defined as semi-random behaviors of number sequences generated by well-tailored nonlinear deterministic systems [27]. As mentioned, many chaos-induced metaheuristic algorithms have been developed in recent years, leveraging the favorable properties of chaotic numbers over randomly generated numbers drawn from a uniform distribution [28]. Beyond this utilization of chaotic sequences, the primary idea is to transform the pseudo-random chaotic numbers into the prescribed solution space. The tedious process of searching global optimum points heavily depends on the intrinsic features of the employed chaotic maps, such as ergodicity, regularity, and stochastic properties. Chaos-enhanced algorithms successfully converge more quickly to the optimal solution and avoid local pitfalls within the solution domain. These favorable and reliable function characteristics put chaotic algorithms one step ahead of their contemporary alternatives.

Contrary to the majority of previous works, which have been based on chaotic metaheuristic algorithms available in the existing literature and have considered only a limited number of chaotic maps for comparative evaluation, this research study utilizes twenty-one non-invertible deterministic chaotic maps to be integrated into the original Mountain Gazelle Algorithm. These twenty-one different chaotic maps have been separately implemented into the standard Mountain Gazelle Optimizer to evaluate the optimization performance improvement made through the merits of the chaotic numbers. A comparative assessment of the solution quality improvement is achieved by analyzing the mean fitness values obtained after running thirty independent algorithms run for each chaotic variant of the Mountain Gazelle Optimizer. The twenty-one distinct chaotic maps separately to be embedded into standard Mountain Gazelles are given as follows: Arnold map (CH01) [29], Bernoulli Map (CH02) [30], Chebyshev Map (CH03) [31], Chirikov Map (CH04) [32], Gauss Map (CH05) [33], Gingerbreadman Map (CH06) [34], Henon Map (CH07) [35], Ikeda Map (CH08) [36], Baker Map (CH09) [37], Iterative Map (CH10) [38], Kent Map (CH11) [39], Logistic Map (CH12) [40], Lozi Map (CH13) [41], Piecewise Map (CH14) [42], Sawtooth Map (CH15) [43], Sinai Map (CH16) [44], Sine Map (CH17) [45], Singer Map (CH18) [46], Standard Map (CH19) [37], Tent Map (CH20) [47], and Zaslavskii Map (CH21) [48].

### Decision Process of the Best-Performing Chaotic Mountain Gazelle Algorithm Variant

This subsection provides a brief explanation of the procedural steps for selecting the best-performing chaotic MGO variant among competing alternatives. As previously described in the above section, chaotic pseudo-number sequences extracted from different chaotic maps are separately substituted into the main running MGO by removing uniformly generated random numbers employed in the original version of the algorithm. Past studies on chaotic metaheuristic approaches have revealed that integrating chaos into the optimization method significantly improves the overall search efficiency of the governing algorithm. Building on previous literature, this study will utilize twenty-one distinct chaotic maps collected from literature studies to enhance the two pivotal yet complementary search mechanisms of exploration and exploitation. This entails a significant boost in convergence rates and improved solution diversity.

**Figure 1:**
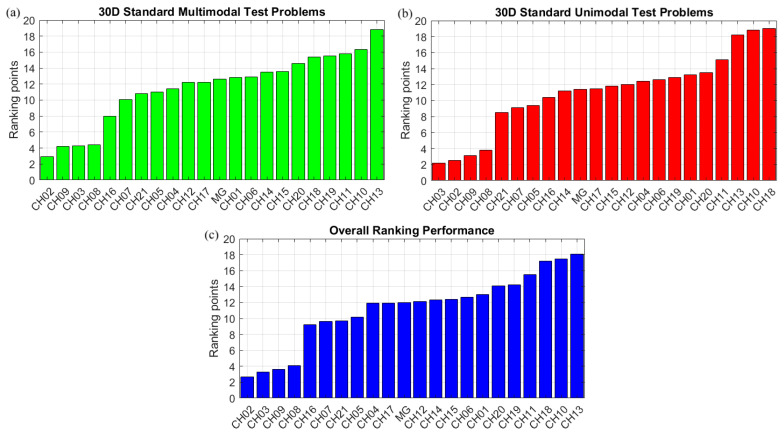
Rankings of the compared chaotic MGO variants based on their mean fitness values obtained from 30 independent runs for 30D test functions: (**a**) multimodal, (**b**) unimodal, and (**c**) overall ranking performance.

**Figure 2:**
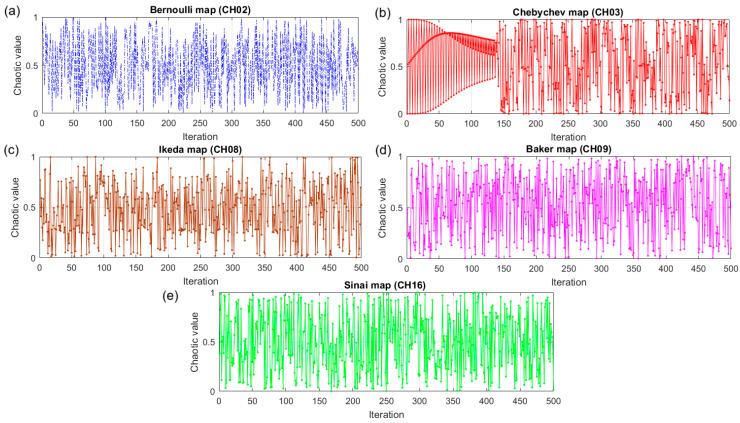
Sequential pseudo-random numbers generated by (**a**) Bernoulli map, (**b**) Chebychev map, (**c**) Ikeda map, (**d**) Baker map, and (**e**) Sinai map.

All MGO-based chaotic algorithms compared are developed in MATLAB 2018 and run on a laptop computer with an Intel Core i7 processor and 16 GB of RAM. Twenty-one chaotic MGO algorithms are benchmarked against each other using forty-eight widely used optimization test functions, comprising 30D multimodal and unimodal artificially generated problems, with functional descriptions in Table 1. Each competitive, chaotic MGO variant has been applied to this pool of challenging test functions. The chaotic MGO with the best mean rank value will be considered for further evaluation. All chaotic methods are independently run 50 times for 2000 function evaluations. Algorithms are ranked according to the mean fitness values obtained for each benchmark problem. The most effective method is the chaotic optimizer with the lowest mean value, based on its cumulative performance averaged over forty-eight distinct optimization test functions. Figure 1 compares the ranking performance of the chaotic algorithms based on their mean fitness values for each test function used. In Figure 1, MG stands for the original Mountain Gazelle algorithm. For 30D multimodal test problems, the CH02 chaotic variant becomes the dominant algorithm for most test cases and ultimately wins the contest for multimodal problems. CH09 is the second-best-performing algorithm for multimodal test problems, yielding relatively persistent estimates for all test cases except those involving the *F*_4_, *F*_15_, and *F*_22_ functions. Although CH02 provides the best mean predictions in 16 out of 24 unimodal test problems, the estimation incompetence exhibited by *F*_26_ and *F*_45_ places this chaotic variant in second place after the CH03 algorithm, which consistently ranks high across the unimodal benchmark suite when the mean results are averaged. Another algorithm demonstrating robust solution consistency is CH09, which suffers from the poor estimation obtained for the *F*_45_ test problem, rendering this algorithm the third-best method for 30D unimodal problems. The overall ranking performance of the compared algorithms, as reported at the bottom part of Figure 1, reveals that CH02 (Bernoulli map) is the most accurate algorithm, with a ranking point of 2.7, which is the average value of the ranking points obtained for multimodal and unimodal test problems in the defined test suite. CH03 (Chebyshev) ranks second with a ranking point of 3.3. CH09 (Baker map), CH08 (Ikeda map), and CH16 (Sinai map) are found to occupy the third, fourth, and fifth positions in the overall performance evaluation of the optimization. Figure 2 illustrates the sequential chaotic numbers generated by the five best-performing algorithms.

## 4. Improved Mountain Gazelle Optimization Algorithm

The algorithm begins with the chaotic Latin Hypercube Sampling mechanism to ensure that initial candidate solutions are well distributed across the multidimensional solution domain, a crucial step in augmenting diversity in the population before subsequent iterations proceed. Then, the iterative process starts with applying chaos-induced MGO to the defined optimization problem. After following the basic steps of the chaotic algorithm, the proposed scheme is activated with a probability of 0.5, thereby avoiding the need to expend extravagant computational resources. The first modified model integrates the Quasi-Oppositional and Dynamic Opposition-Based Learning methods, combining them into a single procedure. It also considers the influences of cosine and sine trigonometric functions to ensure nonlinear and diverse movements, thereby helping to avoid entrapment in local minima. The second modified version of QDOPP proposed in this study utilizes a novel random number generator based on trigonometric functions, founded on the concept of spiraling dynamics and adaptive fitness-driven perturbations, which adjust the direction of the perturbation in response to changes in the fitness landscape. These two intelligently devised perturbation schemes have been guided by a novel adaptive switch mechanism, which simultaneously considers the varying solution diversity in the population, the ratio of the improved solutions in the current population, and the quality of the best fitness value in the evolving populations of competing alternate QDOPP algorithms. The upcoming subsections briefly explain the basic principles underlying these innovative search procedures.

### 4.1. Population Initialization with Chaos-Induced Hybrid Latin Hyper Cube Sampling and Opposition-Based Learning

Many past literature studies emphasize the importance of generating highly diverse population members in the initialization phase, which enables significantly better predictive results during the iterative process. The concept of Opposition-Based Learning has been widely integrated in the population initialization phase to diversify the search space as much as possible [49]. This is a favorable option for initializing candidate solutions with higher qualities, as computing the opposite numbers is a relatively straightforward process that does not excessively consume computational resources. Consider an evolving population *X* with *N*-sized population members, each comprising *D*-dimensional candidate solutions. The opposite point of each trial solution is calculated by

(10) $$X_{i,j}^{OP}={low}_{j}+{up}_{j}-X_{i,j}\;\;\;\;\;\;\;\;\;\;\;i=1,2,3,..,N\;\;\;\;\;j=1,2,3,\ldots ,D$$

where *X^OP^* is the opposite point of each individual in the *X* population, and *low* and *up* represent the lower and upper limits of the *j*th dimension. Another option can be to embed pseudo-random numbers generated by various chaotic maps into the representative model responsible for producing the initial population. Apart from the different learning schemes, each with its intrinsic computational advantages in generating the initial population for metaheuristic algorithms, statistical tools have also been consistently employed in the population initialization process. Among them, the Latin Hypercube Sampling (*LHS*) method is a well-established example of a statistical approach that has proven successful. It has also been one of the most adopted statistical procedures for metaheuristic initialization. This method is an efficient way to produce sample inputs drawn from a multidimensional distribution [24]. It ensures that the generated samples are well scattered across the defined range, resulting in uniform stratification along each dimension and the avoidance of clustering in the population while preserving high diversity. This is much better guided than pure random sampling through the LHS method. However, although this algorithmic procedure is a prolific alternative for generating samples evenly distributed across the dimensions, it may not be advantageous for sampling high-dimensional spaces, as the imposed complexity may not allow for efficiently filling the search space. In addition, the artificially structured stratification of samples can introduce an unexpected correlation between them, jeopardizing the generated randomness. Moreover, despite its effectiveness in covering the entire search span with evenly distributed random numbers, LHS might not be sufficient to probe these critical regions, where promising areas to reach global optimum solutions probably reside. Therefore, to eliminate the procedural drawbacks of the LHS method, this study proposes integrating chaotic sequences generated by the logistic map [40] to achieve enhanced diversity in the population, reduce the correlation between sampled parameters, and avoid entrapment in troublesome local minima during the early phase of iterations. Below, the mathematical model explains how chaotic numbers of the logistic map are introduced into the algorithmic procedure of the Latin Hypercube Sampling method.

Suppose that *X* is an *N*-sized *D*-dimensional population representing the sampled individuals and each member is defined by ${\vec{x}}_{i}=\left(x_{i,1},x_{i,2},x_{i,3},\ldots ,x_{i,D}\right)$. For each different variable of $x_{j}$, the domain of the *i*th individual is divided into *N* equally spaced non-overlapping intervals, expressed by the following equation

(11) $$M_{j}^{k}=\left[\frac{k-1}{N},\;\frac{k}{N}\right],\;\;\;\;\;\;k=1,2,\ldots ,N,\;\;\;\;\;\;j=1,2,3,\ldots ,D$$

The above formulation is a crucial step in the algorithm, specifically in stratifying the sampled parameters, ensuring that at least one sample is placed within the defined interval. Therefore, for each variable *x_j_*, *N* intervals are prepared to provide a uniform marginal distribution. The next step is to generate a random offset within the range of interval sets, using sequential chaotic numbers from the logistic map rather than uniformly distributed Gaussian random numbers. The following equation computes the randomized placement of samples within the interval.

(12) $$r_{j}^{k}=\frac{k-1+{chx}_{j}^{k}}{N},\;\;\;k=1,2,\ldots ,N,\;\;\;j=1,2,3,\ldots ,D$$

where *chx* is a chaotic number ensuring the random offset in the interval, which is generated by the logistic map calculated by the following equation [40].

(13) $${chx}_{k+1}=4\cdot {chx}_{k}\cdot \left(1-{chx}_{k}\right),\;\;\;\;\;k=1,2,\ldots ,N\;$$

The sampling order in each dimension is shuffled to avoid correlation among the generated variables, assuming that ${\vec{C}}_{j}=\left(c_{1}^{j},c_{2}^{j},\ldots ,c_{N}^{j}\right)$ is a random permutation vector of {1, 2, 3, …, *N*} for each *j*. Applying a randomly generated permutation vector of ${\vec{C}}_{j}$ rearranges the stratified samples through the below expression.

(14) $$x_{i,j}=r_{j}^{c_{i}^{j}}\;\;i=1,2,\ldots ,N,\;\;\;\;j=1,2,3,\ldots ,D$$

Finally, stratified random solutions are scaled into the predefined range between *low_j_* and *up_j_* using the equation below.

(15) $$x_{i,j}={low}_{j}+\left({up}_{j}-{low}_{j}\right)\cdot r_{j}^{c_{i}^{j}}\;\;\;\;i=1,2,..,N\;\;j=1,2,3,..,D$$

This equation ensures that each sample remains within its predefined range. The algorithm below explains the basic steps of the proposed hybrid population initialization scheme. MATLAB code of the proposed Latin Hypercube Sampling scheme can be found in the Supplementary Materials (LHS_Logistic.m).

### 4.2. Hybrid Chaotic Quasi-Opposition-Based Learning and Quasi-Dynamic Opposition-Based Learning Search Mechanism

This section examines innovative approaches for integrating Quasi-Opposition-Based Learning [50] (QOBL) and Quasi-Dynamic Opposition-Based Learning [26] (QDOPP) algorithms to enhance solution diversity and prevent convergence stagnation during iterations. There are various Opposition-Based Learning models previously discussed in existing literature. Despite its recent emergence, the Dynamic Opposition-Based Learning search mechanism [51] presents a favorable alternative to the remaining opposition-based variants, which can achieve high-quality solutions within a reasonable runtime comparable to those of the available alternative variants. QOBL is a widely known method that performs perturbations between the center and opposite points to enhance solution quality in the evolving population. However, its overall impact may be limited when the governing search space is irregular or if opposite points do not reflect a plausible symmetry. Additionally, quasi-opposition points may be over-randomized, which puts the algorithm at risk of wasting unnecessary function evaluations on regions that are not fertile. Past experiences with the probing efficiency of QDOPP reveal that similar algorithmic drawbacks evident for QOBL are also applicable to QDOPP. The success of QDOPP in the search heavily depends on the spatial structure of the search space. Therefore, if dynamic adjustments are performed too aggressively, the algorithm tends to delay convergence due to exploring irrelevant areas. Furthermore, the adverse effects of the curse of dimensionality hinder the generation of asymmetric sample points in highly irregular problems, which are prevalent in many real-world optimization problems. These two methods are integrated into a single scheme to alleviate the algorithm-specific disadvantages of the QDOPP and QOBL search strategies. Trigonometric sine and cosine functions are also embedded into this proposed scheme in the random number generation stage. Employing sine and cosine functions introduces a periodic and smooth variation between exploration and exploitation phases through controlled oscillations in the search space, which is also beneficial in avoiding premature convergence. In addition to these features, a structured non-uniform distribution created by sine and cosine functions, when synergized with an iteration counter or other time-varying parameters, can help focus on fertile regions more effectively. Finally, chaotic numbers generated by the Chebyshev map are integrated into this manipulation procedure to enhance the contributions of the above-defined improvements, aiming to boost population diversity and eliminate local pitfalls in the search space. A simple yet effective formulation of the Chebyshev chaotic map [31] is given below.

(16) $${chx2}_{t+1}=cos\left(t\cdot arccos(ch{x2}_{t})\right)$$

where *chx*2 is a chaotic number defined in the range 0 and 1; *t* represents the current iteration; and *t* + 1 stands for the next iteration. When t≥2, chaotic behaviors are observed with increasing iterations. The Quasi-Dynamic Opposition Learning and Quasi-Opposition-Based Learning search mechanisms are provided below.

Let $\vec{X}=\left(x_{1},x_{2},\ldots ,x_{D}\right)$ represent a vector in a *D*-dimensional space and $x_{j\;}\in [{low}_{j},{up}_{j}]$ where *j* = 1, 2, …, *D*, and *low* and *up* correspondingly stand for the lower and upper limits of the search space. The quasi-opposite solution (*X^QOBL^*) can be expressed in D-dimensional space by the following.

(17) $$X_{j}^{QOBL}=rand\left(\;\;0.5\left({low}_{j}+{up}_{j}\right),\;\;\;{low}_{j}+{up}_{j}-X_{j\;\;}\right)$$

In the above equation, *rand*(a,b) generates a uniform random number between a and b. Similarly, the Dynamic Opposite Point (*X^DOPP^*) in a D-dimensional space is calculated by using the following set of equations.

(18) $$X_{j}^{OP}={low}_{j}+{up}_{j}-X_{j\;\;}$$

(19) $$X_{j}^{DOPP}=X_{j}+\omega \cdot {rand}_{1}(0,1)\cdot \left({rand}_{2}\left(0,1\right)\cdot X_{j}^{OP}-X_{j}\right)$$

where ω is a weight factor, and *rand*_1,2_(0,1) are two random numbers between 0 and 1 drawn from a uniform distribution. The algorithm below outlines the procedural steps for integrating these two opposing learning-based variants and synergizing the trigonometric functions of sine and cosine with chaotic numbers generated from the Chebyshev map.

The algorithmic procedure between lines 6 and 11 explains the integration of the QOBL and DOPP learning methods. As seen from Algorithm 1, the governing equation of DOPP (Line 8 and Line 11) uses the Quasi-Opposite solution rather than the Opposite points—*X^OP^*. This preference offers several advantages to achieving high solution efficiency. Increased population diversity is one of the significant consequences of this integration since the adaptive opposition of DOPP enables the preservation of diversity at early stages. At the same time, the center-based solution generation of QOBL maintains diverse solutions in the latest iterations. Another advantage is the improved robustness across various problem types, as different problems require different search characteristics. For instance, DOPP can be considered a favorable alternative search mechanism for complex and rugged fitness landscapes, while QOBL’s local search characteristics render this learning method suitable for smooth problems. That is to say, the dynamic nature of the DOPP algorithm allows search agents to perform intense exploration. At the same time, the alternative use of QOBL enables a smooth shift between exploration and exploitation, counterbalancing the contradictory search mechanisms. In addition, trigonometric sine and cosine functions provide smooth transitions between values of −1 and 1, enabling fine-grained control over generating opposites and eliminating the risk of generating opposite points that fall outside a reasonable range. Integrating Chebyshev map-based chaotic numbers with trigonometric functions significantly enhances the impact of these advantages, which is conducive to avoiding premature convergence and providing better coverage of the search space. Between lines 15 and 17 in Algorithm 2, the population individuals of *X^QOBL^* and *X^DOPP^* are combined into a new *X^ALL^* population. Infeasible solutions in *X^ALL^* are amended through the responsible boundary check mechanism, and the fittest N members are selected and stored in *X^BESTN^* population to be evaluated in upcoming iterations. The respective MATLAB code of the developed Hybrid Opposition-Based Learning procedure is provided in the Supplementary Materials (HOBL.m).
**Algorithm 1:** Hybrid population initialization schemeInputs: Population size–*N*, Problem dimension–*D*,      Upper and lower limits of the search space (*up* and *low*)      Initialize: *N*-sized *D*-Dimensional *X* population defined within the search limits      Employ: logistic map induced LHS to generate evenly distributed population members          *X_LHS_* = LHS (*low*, *up*, *N*, *D*, *chx*)      Produce: opposite points (*X^OP^*) of *X* population using Equation (10)      Combine: population individuals of *X*, *X^OP^*, and *X_LHS_*          and select the fittest N solutions (*X_BESTN_*) between the competitive candidatesOutput: The best N solutions (*X_BESTN_*) to be used for the iterative process
**Algorithm 2:** Hybrid Opposition-Based Learning Procedure (HOBL)Inputs: Evolving population—*X*; Chebyshev chaotic numbers—*chx*       Lower and upper limits of the search space (*low* and *up*) 1     [N, D] = size (X) 2     **for**
*i* = 1 to N 3         **for**
*j* = 1 to D 4          $X_{i,j}^{OP}={low}_{j}+{up}_{j}-X_{i,j}$
 5            $C_{j}=0.5\cdot \left({low}_{j}+{up}_{j}\right)$
 6            **if**
$X_{i,j}<C_{j}$
 7              $\;X_{i,j}^{QOBL}=C_{j}+\left(X_{i,j}^{OP}-C_{j}\right)\cdot sin\left({chx}_{i,j}^{1}\right)$
 8              $X_{i,j}^{DOPP}=X_{i,j}+sin\left({chx}_{i,j}^{2}\right)\cdot \left({chx}_{i,j}^{3}\cdot X_{i,j}^{QOBL}-X_{i,j}\right)$
 9            **else** 10             $\;X_{i,j}^{QOBL}=X_{i,j}^{OP}+cos\left({chx}_{i,j}^{4}\right)\cdot \left(C_{j}-X_{i,j}^{OP}\right)$
 11             $X_{i,j}^{DOPP}=X_{i,j}^{QOBL}+cos\left({chx}_{i,j}^{5}\right)\cdot \left({chx}_{i,j}^{6}\cdot X_{i,j}-X_{i,j}^{QOBL}\right)$
 12             **end** 13         **end** 14    **end** 15        *X^ALL^* = [*X^QOBL^*;*X^DOPP^*] // Combine two populations 16        *X^ALL^* = boundary (*X^ALL^*) // Apply boundary check mechanism 17        *X*^BESTN1^ = sort (*X^ALL^*,1:*N*)   // Select the fittest N individualsOutput: *X*^BESTN1^


### 4.3. Hybridization of Quasi-Dynamic Opposite Learning Search Mechanism (QDOPP) with a Novel Trigonometric Random Number Generator and Adaptive Fitness-Based Perturbation Scheme

This section addresses the algorithmic drawbacks of Quasi-Dynamic Opposition-Based Learning by introducing a novel trigonometric random number generator and an adaptive search equation guided by the respective fitness values of population individuals. Although the effectiveness of the QDOPP algorithm was verified over various benchmark cases of unconstrained and constrained high optimization problems, evident shortcomings still prevail, such as potential over-exploration caused by the concurrent utilization of dynamic adjustments and quasi-opposite numbers. This may lead to problematic outcomes where solution refinement in near-optimal areas is more crucial and necessary. Similar to most opposition-based algorithms, QDOPP also suffers from problem dependency and fitness landscape characteristics; that is, it may excel in problems with multimodal characteristics but may underachieve in more straightforward convex cases. Furthermore, generating opposite numbers to achieve promising solutions can be meaningless for high-dimensional problems due to inefficient exploration. Therefore, this section proposes a novel trigonometric random number generator with numerous functional advantages to address the evident drawbacks of QDOPP. A brand-new Inverse Sinusoidal Spiral Map-Based Random Number Generator (ISSM-RNG) is proposed in this section, which facilitates the generation of diverse yet stable random numbers. This method leverages the functional characteristics of inverse sinusoidal functions, generating spiraling dynamics that evolve chaotic yet bounded sequences. The following equation generates a recursive sequence of random numbers.

(20) $$r_{t+1}=\left|sin\left({2\cdot r}_{t}\right)+1.5\cdot cos\left({3\cdot r}_{t}^{2}\right)+2.2\cdot sin\left({1.8\cdot r}_{t-1}\right)+2.6\cdot cos\left(1.9\cdot r_{t}\cdot r_{t-1}\right)+\frac{1}{1+r_{t}^{2}}\right|\;\;\;mod\;1$$

**Figure 3:**
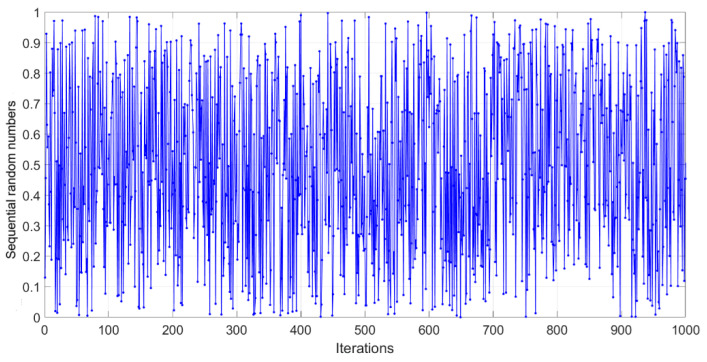
Random numbers bounded between 0 and 1 generated by ISSM-RNG.

The inverse function $1/\left(1+r_{t}^{2}\right)$ in Equation (20) introduces spiral shrinkage, preventing runaway values in the consecutive sequence. The interaction between the sine and cosine functions incorporates oscillatory actions that promote diversity in the sequential random numbers. The sequence is bounded between 0 and 1 by using the mod 1 function. This random number generation method can be beneficial for utilizing metaheuristic algorithms, as the dynamic spirals result from combining a sinusoidal term and an inverse function, which helps diversify the search space. Additionally, the inverse term prevents the system from diverging rapidly and prematurely. Furthermore, integrating the previous random number (*r_t_*_−1_) into the model introduces a memory effect, effectively improving adaptability. Figure 3 shows the dynamically updated random values produced by the ISSM-RNG. The MATLAB code of this sequential random number generation procedure is given in the Supplementary Materials (ISSM_RNG.m).

Another novelty proposed in this section is the introduction of the adaptive fitness-driven perturbation (AFDP) strategy. This parameter-free scheme significantly enhances the diversity of the general population in metaheuristic optimizers. The main idea of this concept relies on adjusting the magnitude and adaptation of perturbations based on the variation in fitness values among population members. The self-regulation nature of this algorithmic procedure does not necessitate predefined parameters, and the fitness difference between the best and ordinary members guides the magnitude of the perturbations. In this context, fitter individuals undergo more minor perturbations to avoid over-exploration, while less fit members experience more significant perturbations to improve diversification. The equation below defines the fundamental equation of the fitness-driven perturbation scheme, which incorporates diversity and different probability distribution functions.

Assume that the population *X* is comprised of *N*-sized *D*-dimensional candidate solutions represented by $X=\left(x_{1},x_{2},\ldots ,x_{N}\right),\;x_{i}\in R^{D}$. The objective function of the problem can be expressed by $f:R^{D}\to R$, where the best fitness in the population is $f_{best}=\min f(x_{i})$.

For each trial solution in population *X*, the normalized fitness gap is calculated by

(21) $$\delta (x)=\frac{f(x)-f_{best}}{max\left(\left|f(x)\right|,\left|f_{best}\right|\right)+\epsilon }$$

where ϵ is a small positive constant very close to zero, used to avoid the possibility of division by zero. The proposed scheme considers the current diversity in the population to define an adaptive scale among individuals, calculated as follows.

(22) $$Diver=\frac{1}{D}\sum_{j=1}^{D}\left(\frac{1}{N}\sum_{i=1}^{N}{\left(x_{i,j}-\bar{x_{j}}\right)}^{2}\right)\mathrm{where}\;\bar{x_{j}}=\frac{1}{N}\sum_{i=1}^{N}x_{i,j}\;\;\;j=1,2,\ldots ,D$$

An adaptive perturbation scale is introduced to the algorithm, coupling the fitness gap with the current diversity.

(23) σ(x)=δ(x)·Diver

This mathematically explains that the trial solution, which is farther from the optimal solution (larger *δ*(*x*)) in a more diverse population (higher *Diver_pop_*), experiences higher perturbation. In contrast, members starting to converge on optimal solutions have comparatively lower fitness gap values and diversity rates, promoting local exploitation. The perturbed individual is calculated by

(24) $$x_{new,i}=x_{i}+\sigma (x)\cdot {vec}_{rand}(x)\;i=1,2,\ldots ,N$$

where ${vec}_{rand}(x)$ is a random perturbation vector drawn from alternative probability distributions whose shape is decided by the quality of the online performance feedback. The adaptive scores related to solution improvement rates determine the probability of switching between Gaussian, Cauchy, or Levy distributions. This adaptive distribution operator selection is described in the following.

Assume a set of operation operators is available to algorithm O = {*o*_1_, *o*_2_, …, *o_k_*}. For each operator o in O, a respective performance score is assigned to *S_o_*(*t*) and updated in each iteration. Consider an operator *o* chosen from available candidates employed at iteration *t*, which improves solution quality, is computed by

(25) $$\Delta f_{o,i}=f(x_{i})-f(x_{new,i})\;i=1,2,\ldots ,N$$

where $f(x_{i})$ and $f(x_{new,i})$ are, respectively, the objective function values of the current and updated *i*th member of the population. Then, its respective performance score is updated using the following formula.

(26) $$S_{o}^{t+1}=S_{o}^{t}+{\Delta f}_{o,i}^{t}-P_{o}^{t}\;i=1,2,\ldots ,N$$

where $S_{o}^{t}$ is the current performance score when the distribution operator *o* is employed, ${\Delta f}_{o,i}^{t}$ is the fitness improvement observed when the perturbation operator *o* is applied to the *i*th member at iteration *t*, and $P_{o,i}^{t}$ is the penalization factor when fitness improvement is not observed by the application of the distribution operator *o*. Then, the probabilistic selection of the distribution operator *o* valid for iteration *t* is calculated by

(27) $${pr}_{o,i}^{t}=\frac{S_{o,i}^{t}}{\sum_{n\in O}S_{n,i}^{t}}$$

This probabilistic selection equation enables the algorithm to self-adjust the considered perturbation schemes without using preset constant parameters. Algorithm 3 provides the algorithmic steps of the proposed adaptive fitness-driven perturbation model.

The MATLAB code of the Adaptive Fitness-Driven Perturbation scheme can be found in the Supplementary Materials (AFDP.m).**Algorithm 3:** Adaptive Fitness-Driven Perturbation algorithm—AFDPInputs: Population members—*X*; objective function—*f_obj_* ()        [*N*,*D*] = size (*X*)  // Determine the population size N and problem dimension D        Calculate: the diversity of the population *Diver* using Equation (22)        Decide: the best individual among the current population—*f*_best_        Initialize: the operator performance scores–*S*_o_        **for** i = 1 to N          // each population member in *X*           *Calculate*: the normalized fitness gap $\delta (x_{i})$
using Equation (21)           *Compute:* the adaptive scale factor $\;\sigma \left(x_{i}\right)$
using Equation (23)           *Determine*: the operator selection probability–*pr_o_*
                      from the current scores using Equation (27)           **if**
*rand*(0,1) < *pr*_o_// Randomly select the operator according to *pr_o_             *             o = 1           // Consider perturbation based on Gaussian distribution 
             vec_rand_ (*x_i_*) = Gaussian(1,D)           **else**             o = 2           // Consider perturbation based on Cauchy distribution             vec_rand_ (*x_i_*) = Cauchy(1,D)           **end**           *Obtain*: the updated population member *x_new_*_,i_ using Equation (24)           *Perform*: boundary check on *x_new_*_,i_ to verify solution feasibility           *Evaluate*: the fitness value of *x_new_*_,*i*_ − *f_obj_*(*x_new_*_,*i*_)           **if**
*f_obj_* (*x_new_*_,*i*_) < *f_obj_* (*x_i_*)     // Accept candidate if improvement is observed             *x_i_* = *x_new_*_,*i*_             Δ*_i_* = *f_obj_* (*x_i_*) − *f_obj_* (*x_new_*_,*i*_)  // Employ Equation (25) to calculate improvement             *S_o_* = *S_o_* + Δ*_i_*      // Update the operator score (*S_o_*)           **else**             *S_o_* = *S_o_* • 0.99 // *P_o_*—Penalize the operator if no improvement occurs           **end**        **end**Output: *X^UPTD^*—updated population members

The proposed algorithm, founded upon the hybridization of the ISSM-RNG-induced QDOPP and Adaptive Fitness Driven Perturbation Scheme, takes the final form, which is explained in Algorithm 4. **Algorithm 4:** Hybrid ISSM-RNG induced QDOPP–AFDP algorithm (AFD-QDOPP)        Inputs: Population individuals—*X*; objective function—*f_obj_* ()              Lower and upper bounds (*low* and *up*) 1       [*N*, *D*] = size (*X*) 2       Generate: *N*-sized *D*-dimensional random number sequences using ISSM-RNG 3       **for**
*i* = 1 to *N* 4          **for**
*j* = 1 to *D* 5            $X_{i,j}^{OP}={low}_{j}+{up}_{j}-X_{i,j}$
6            $C_{j}=0.5\cdot \left({low}_{j}+{up}_{j}\right)$
7            **if**
$X_{i,j}<C_{j}$
8              $X_{i,j}^{QDOPP}=X_{i,j}+{SRN}_{i,j}^{1}\cdot \left({SRN}_{i,j}^{2}\cdot X_{i,j}^{OP}-X_{i,j}\right)$
9            **else** 10              $X_{i,j}^{ROP}={SRN}_{i,j}^{3}\cdot X_{i,j}^{OP}$
11                $X_{i,j}^{QDOPP}=X_{i,j}^{ROP}+{SRN}_{i,j}^{4}\cdot \left({SRN}_{i,j}^{5}\cdot X_{i,j}-X_{i,j}^{ROP}\right)$
12             **end** 13          **end** 14       **end**
15       *Employ*: boundary check mechanism to repair infeasible solution in *X^QDOPP^* 16       *X^UPDT^* = AFDP (*X^QDOPP^*, *f_obj_*) 17       *Amend*: the violated solutions in *X^UPDT^* 18       *X^ALL^*^2^ = [*X^QDOPP^*; *X^UPDT^*] 19       *X*^BESTN2^ = sort (*X^ALL^*^2^,1:*N*) 20       Output: *X*^BESTN2^

In Algorithm 4, ISSN-RNG-based random number generation is repeated five times (Line 2) to be integrated into QDOPP, whose fundamental search equations are performed between lines 3 and 14. The boundary check mechanism is activated in Line 15 to repair feasible solutions in *X^QDOPP^*. The AFDP, as described in Algorithm 3, is run for the input values of *X^QDOPP^* to enhance the overall solution quality within the population. Following that, a boundary control scheme is employed for the newly generated solutions of *X^UPDT^*. Two different N-sized populations, *X^QDOPP^* and *X^UPDT^*, are combined in *X^ALL^*^2^ and sorted according to their fitness values. The N-fittest individuals among them are stored in *X^BESTN^*^2^. The MATLAB code for the QDOPP-AFDP algorithm is available in the Supplementary Materials (QDOPP_AFDP.m).

### 4.4. Majority Voting Adaptive Switch Mechanism

This study proposes a novel parameter-free adaptive switch mechanism to determine which of the proposed procedures in Algorithm 2 or Algorithm 4 to use in the ongoing iterative process. An adaptive shift addresses the intrinsic challenges of deciding which algorithm to apply between the two methods during consecutive iterations, considering the inherent diversity, best fitness quality, and total fitness improvement in the evolving population. This intelligently devised model makes timely decisions about when to switch between two competing algorithms, employing a democratic voting system that evaluates the current situation of the population without making any assumptions or using preset constant parameters. The proposed mechanism evaluates three primary votes based on the changes in the state metrics. The model switches to the other algorithm if at least two out of three votes suggest the change. At each iteration after the initialization, the algorithm computes the current diversity of the population *Diver*, which can be calculated by Equation (22); the best fitness in the population *fbest*; and the improvement in best fitness value *I_t_*, which is calculated by

(28) $$I_{t}=max\left(0,{fbest}_{t-1}-{fbest}_{t}\right)$$

In this respect, a positive value of It indicates progress (if function minimization is considered), while zero indicates no further progress, leading to stagnation. This adaptive switch mechanism utilizes the Moving Weighted Average (MWA) of each metric, which is iteratively updated based on its current and previous values. For each different metric, MWA updates itself using the following equations.

(29) $${MWA}_{Diver}^{t}={\alpha_{t}\cdot Diver}_{t}+\left(1-\alpha_{t}\right)\cdot {MWA}_{Diver}^{t-1}$$

(30) $${MWA}_{fbest}^{t}={\alpha_{t}\cdot fbest}_{t}+\left(1-\alpha_{t}\right)\cdot {MWA}_{fbest}^{t-1}\;\;$$

(31) $${MWA}_{I}^{t}={\alpha_{t}\cdot I}_{t}+\left(1-\alpha_{t}\right)\cdot {MWA}_{I}^{t-1}$$

In the above equations, a smoothing factor *α* is incorporated that gives importance to the recent data rather than the previous one, balancing responsiveness and stability. This time-varying model parameter α is calculated by the following expression:

(32) $$\alpha_{t}=\alpha_{start}-\left(\alpha_{start}-\alpha_{end}\right)\frac{t}{Maxiter}$$

In Equation (32), $\alpha_{start}$ and $\alpha_{end}$ are initial and final values of the iterative $\alpha_{t}$ parameter, respectively, assigned to 0.5 and 0.1; *t* is the current iteration, while *Maxiter* is the total number of iterations required to terminate the iterative process.

The model accounts for variations in trends by calculating the differences between consecutive MWA values obtained for different metrics.

(33) $${\Delta MWA}_{Diver}^{t}={MWA}_{Diver}^{t}-{MWA}_{Diver}^{t-1}$$

(34) $${\Delta MWA}_{fbest}^{t}={MWA}_{fbest}^{t}-{MWA}_{fbest}^{t-1}$$

(35) $${\Delta MWA}_{I}^{t}={MWA}_{I}^{t}-{MWA}_{I}^{t-1}$$

These sequential differences represent the current situation of whether each metric is improving or worsening. If ${\Delta MWA}_{Diver}^{t}<0$, it means that diversity is decreasing and the population is converging. If ${\Delta MWA}_{fbest}^{t}>0,$ it indicates that fitness quality is worsening, suggesting that the switch between two algorithms is necessary. If ${\Delta MWA}_{I}^{t}<0$, the improvement in fitness rates is slowing down. Then, the voting mechanism is put into practice, in which each different metric casts a binary vote to determine whether to switch (1) or stay (0), which prevails based on the variations in their current trends.

(36) $${Vote}_{Diver}^{t}=\left\{\begin{array}{c} 1\;\;\;\;\;\;\;\;\;\;\;\;if\;{\Delta MWA}_{Diver}^{t}<0\\ 0\;\;\;\;\;\;\;\;\;\;\;\;\;\;\;\;\;\;\;\;\;\;\;\;\;\;\;\;\;otherwise \end{array}\right.$$

(37) $${Vote}_{fbest}^{t}=\left\{\begin{array}{c} 1\;\;\;\;\;\;\;\;\;\;\;\;\;\;if\;{\Delta MWA}_{fbest}^{t}>0\\ 0\;\;\;\;\;\;\;\;\;\;\;\;\;\;\;\;\;\;\;\;\;\;\;\;\;\;\;\;\;\;\;\;otherwise \end{array}\right.$$

(38) $${Vote}_{I}^{t}=\left\{\begin{array}{c} 1\;\;\;\;\;\;\;\;\;if\;{\Delta MWA}_{I}^{t}<0\\ 0\;\;\;\;\;\;\;\;\;\;\;\;\;\;\;\;\;otherwise \end{array}\right.$$

The switching decision occurs if ${Vote}_{Diver}^{t}+{Vote}_{fbest}^{t}+{Vote}_{I}^{t}$ ≥ 2, otherwise the current one survives into the next iteration.

### 4.5. Hybrid Chaos Induced Integrated Quasi-Dynamic Opposition-Based Learning (HCQDOPP)-Enhanced Mountain Gazelle Optimizer

This research study aims to integrate various perturbation schemes with different characteristics to improve the Mountain Gazelle Optimizer’s overall optimization capability. Initially, the logistic chaotic map, integrated with the Latin Hypercube Sampling method and augmented by the contributions of the Opposition-Based Learning strategy, was utilized in the population initialization phase to diversify the search space before the iterative process commenced. Then, random numbers generated by a uniform distribution used in the search equations of MGO are replaced with chaotic numbers from the Bernoulli map to balance the exploration and exploitation phases more effectively and avoid premature convergence. Two different improved Quasi-Dynamic Opposition-Based Learning variants have been proposed to overcome the algorithmic disadvantages of the MGO algorithm, one of which is compromised search quality when problem dimensionalities are increased, and the other is that the algorithm may exhibit slow convergence characteristics when exploring complex spaces. This may occur due to the imbalance between competing search mechanisms that guide the search process. This can sometimes delay the focus on promising regions and promote exploration, or vice versa. In addition, although the assertive claim in the reference paper of the MGO is that there is a proper balance between the exploration and exploitation phases maintained by the conducive communication between the intelligently devised search mechanisms, the fact of the matter is that the algorithm lacks a solution refinement when it is needed to probe around the areas near to the global optimum. It is also observed that MGO may underperform on problems with deceptive and highly constrained search spaces, where gazelle-inspired search strategies are prone to collapse and do not get on well with the structure of the search region. These algorithmic drawbacks of MGO hinder the ongoing probing process to identify fertile areas where the global optimum may reside. The primary motivation behind proposing these complementary and improved QDOPP search mechanisms is to address the deficiencies of MGO and extend its optimization success to a wide range of optimization problems. However, a tedious problem arises as to which algorithm should be used instead of the other one during the iterations, which is resolved by the majority voting adaptive switch mechanism that considers the overall population diversity, total fitness improvement, and variations of the current best fitness values in the population to decide a reasonable selection between two improved QDOPP algorithms. The HCQDOPP-improved MGO algorithm repeats the above-defined manipulation scheme until the predefined termination criterion is met. Algorithm 5, as defined below, explicitly explains the basic algorithmic steps of the proposed method in pseudo-code form.

**Figure 4:**
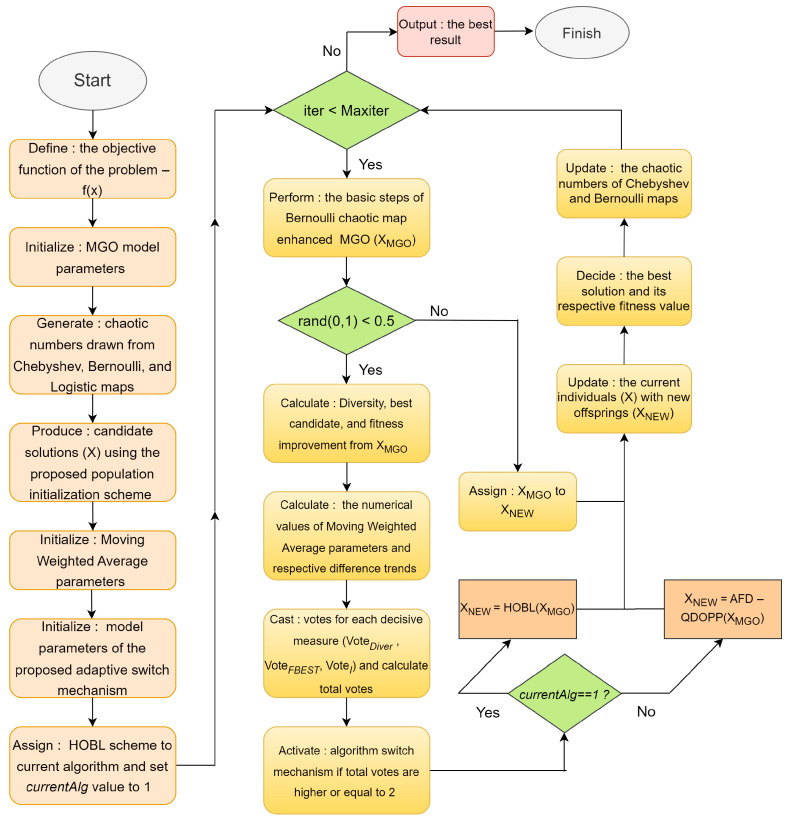
Flow chart representation of HCQDOPP−enhanced MGO algorithm.

Figure 4 shows the algorithmic flowchart representation of the HCQDOPP improved MGO optimizer. **Algorithm 5:** HCQDOPP enhanced MGO optimizerInputs: Objective function—*f_obj_*(); problem size—*N*; problem dimension—*D*       Upper and lower bounds (*up* and *low*), maximum number of iterations (*Maxiter*)       *Initialize*: evolvable population *X* using Algorithm 1       *Initialize*: the model parameters of the running algorithms       *Initialize:* the responsible metrics for the adaptive switch mechanism       *Calculate:* the population diversity (*Diver*) using Equation (22)       *Decide*: the best individual (*X_BEST_*) and its respective fitness value *fbest*       *Set:* the current fitness improvement to zero (*I* = 0)       *Initialize:* Moving Weighted Average parameter values defined for each metric               *MWA_Dive_*_r_ = *Diver*, *MWA_fbest_* = *fbest*, *MWA_I_* = *I*       *Initialize*: chaotic numbers generated by the Chebyshev, Bernoulli, and logistic maps       Assign: HOBL to the current switchable procedure and set currentAlg = 1       *Set:* iteration counter to 1 (*iter* = 1)       **While** (*iter* ≤ Maxiter) do         Run: Bernoulli map improved MGO algorithm           [*X_MGO_*, *X_BEST_*, *fbest*] = MGO(*X*)         **if** rand(0,1) < 0.5             Calculate: Population diversity using *X_MGO_* through Equation (22)                *Diver_iter_* = Diversity (*X_MGO_*)            Use: *fbest* to decide on fitness quality                *fbest_iter_* = *fbest*            Compute: Fitness improvement *I* through Equation (28)                *I* = *if iter* > 1 ? *max*(0, *fbest_prev_* − *fbest_ite_*_r_): 0           Apply: Equation (32) to calculate the numerical value of smoothing factor α_iter_           Update: *MWA_Dive_*_r_, *MWA_fbest_*, *MWA_I_* using Equation (29) to Equation (31) ${\;\;\;\;\;\;\;\;\;\;\;\;\;\;\;\;\;\;\;\;\;\;\;\;\;\;\;\;\;\;\;\;\;\;\;\;\;\;\;\;\;\;\;\;\;\;\;\;\;\;\;\;\;\;\;\;\;\;\;\;\;\;\;\;MWA}_{Diver}^{iter}={\alpha_{iter}\cdot Diver}_{iter}+\left(1-\alpha_{iter}\right)\cdot {MWA}_{Diver}^{prev}$
${\;\;\;\;\;\;\;\;\;\;\;\;\;\;\;\;\;\;\;\;\;\;\;\;\;\;\;\;\;\;\;\;\;\;\;\;\;\;\;\;\;\;\;\;\;\;\;\;\;\;\;\;\;\;\;\;\;\;\;\;\;\;\;MWA}_{fbest}^{iter}={\alpha_{iter}\cdot fbest}_{iter}+\left(1-\alpha_{iter}\right)\cdot {MWA}_{fbest}^{prev}$
${\;\;\;\;\;\;\;\;\;\;\;\;\;\;\;\;\;\;\;\;\;\;\;\;\;\;\;\;\;\;\;\;\;\;\;\;\;\;\;\;\;\;\;\;\;\;\;\;\;\;\;\;\;\;\;\;\;\;\;\;\;\;\;MWA}_{I}^{iter}={\alpha_{iter}\cdot I}_{iter}+\left(1-\alpha_{iter}\right)\cdot {MWA}_{I}^{prev}$
           Compute: the difference trends by using Equation (33) to Equation (35) ${\;\;\;\;\;\;\;\;\;\;\;\;\;\;\;\;\;\;\;\;\;\;\;\;\;\;\;\;\;\;\;\;\;\;\;\;\;\;\;\;\;\;\;\;\;\;\;\;\;\;\;\;\;\;\;\;\;\;\;\;\;\;\;\Delta MWA}_{Diver}^{iter}=if\;iter>1?{\;MWA}_{Diver}^{iter}-{MWA}_{Diver}^{prev}:0$
${\;\;\;\;\;\;\;\;\;\;\;\;\;\;\;\;\;\;\;\;\;\;\;\;\;\;\;\;\;\;\;\;\;\;\;\;\;\;\;\;\;\;\;\;\;\;\;\;\;\;\;\;\;\;\;\;\;\;\;\;\;\;\;\Delta MWA}_{fbest}^{iter}=if\;iter>1?{\;MWA}_{fbest}^{iter}-{MWA}_{fbest}^{prev}:0$
${\;\;\;\;\;\;\;\;\;\;\;\;\;\;\;\;\;\;\;\;\;\;\;\;\;\;\;\;\;\;\;\;\;\;\;\;\;\;\;\;\;\;\;\;\;\;\;\;\;\;\;\;\;\;\;\;\;\;\;\;\;\;\;\Delta MWA}_{I}^{iter}=if\;iter>1?{\;MWA}_{I}^{iter}-{MWA}_{I}^{prev}:0$
           Cast: votes for each decisive metric ${\;\;\;\;\;\;\;\;\;\;\;\;\;\;\;\;\;\;\;\;\;\;\;\;\;\;\;\;\;\;\;\;\;\;\;\;\;\;\;\;\;\;\;\;\;\;\;\;\;\;\;\;\;\;\;\;\;\;\;\;\;\;\;\;\;\;\;Vote}_{Diver}^{iter}=if\;{\Delta MWA}_{Diver}^{iter}<0\;?1\;\;:0$

$\;\;\;\;\;\;\;\;\;\;\;\;\;\;\;\;\;\;\;\;\;\;\;\;\;\;\;\;\;\;\;\;\;\;\;\;\;\;\;\;\;\;\;\;\;\;\;\;\;\;\;\;\;\;\;\;\;\;\;\;\;\;\;\;\;\;\;{Vote}_{fbest}^{iter}=if{\;\Delta MWA}_{fbest}^{iter}>0\;?1\;\;:0$

$\;\;\;\;\;\;\;\;\;\;\;\;\;\;\;\;\;\;\;\;\;\;\;\;\;\;\;\;\;\;\;\;\;\;\;\;\;\;\;\;\;\;\;\;\;\;\;\;\;\;\;\;\;\;\;\;\;\;\;\;\;\;\;\;\;\;\;{Vote}_{I}^{iter}=if{\;\;\Delta MWA}_{I}^{iter}<0\;?1\;\;:0$
             Calculate: the total votes $\;\;\;\;\;\;\;\;\;\;\;\;\;\;\;\;\;\;\;\;\;\;\;\;\;\;\;\;\;\;\;\;\;\;\;\;\;\;\;\;\;\;\;\;\;\;\;\;\;\;\;\;\;\;\;\;\;\;\;\;\;\;\;\;\;\;\;{Vote}_{total}^{iter}={Vote}_{Diver}^{iter}+{Vote}_{fbest}^{iter}+{Vote}_{I}^{iter}$
             Activate: the switching mechanism if necessary conditions are met $\;\;\;\;\;\;\;\;\;\;\;\;\;\;\;\;\;\;\;\;\;\;\;\;\;\;\;\;\;\;\;\;\;\;\;\;\;\;\;\;\;\;\;\;\;\;\;\;\;\;\;\;\;\;\;\;\;\;\;\;\;\;\;\;\;\;\;currentAlg=if\;{Vote}_{total}^{iter}\;$
≥ 2 ? 3–*currentAlg*             Store: the previous MWA metric values and *fbest* for the upcoming iteration ${{\;\;\;\;\;\;\;\;\;\;\;\;\;\;\;\;\;\;\;\;\;\;\;\;\;\;\;\;\;\;\;\;\;\;\;\;\;\;\;\;\;\;\;\;\;\;\;\;\;\;\;\;\;\;\;\;\;\;\;\;\;\;\;\;\;\;\;MWA}_{Diver}^{prev}=MWA}_{Diver}^{iter},\;{{MWA}_{fbest}^{prev}=MWA}_{fbest}^{iter}$
, $\;\;\;\;\;\;\;\;\;\;\;\;\;\;\;\;\;\;\;\;\;\;\;\;\;\;\;\;\;\;\;\;\;\;\;\;\;\;\;\;\;\;\;\;\;\;\;\;\;\;\;\;\;\;\;\;\;\;\;\;\;\;\;\;\;\;\;\;{{MWA}_{I}^{prev}=MWA}_{I}^{iter}$
,   *fbest_prev_* = *fbest_iter_*             Apply: the current algorithm according to *currentAlg* value                **if** currentAlg == 1 then                 *X_NEW_* = HOBL(*X_MGO_*)                **else**                 *X_NEW_* = AFD-QDOPP(*X_MGO_*) **                end**           **else**            Assign: *X_MGO_* to *X_NEW_*        **end**            Activate: boundary search mechanism            Update: *X* population from the newly generated members of *X_NEW_*            Determine: the best solution vector *X_BEST_* and its respective fitness value *fbest*            Update: chaotic sequences generated by Bernoulli and Chebyshev maps            Increment: iteration counter (*iter* = *iter* + 1)        **end****Output:** *X_BEST_*, *fbest*

### 4.6. Time Complexity of the HCQDOPP Algorithm

This section evaluates the total time complexity of the proposed enhanced HCQDOPP algorithm. Although the integration of chaotic numbers increases the total runtime of the algorithm, it does not introduce extra complexity to the algorithmic procedure. Significant complexity differences between the standard MGO and HCQDOPP algorithms stem from the respective position update mechanisms of the two variants, which are utilized in the improvement process of the standard QDOPP algorithm. In the first step, the complexity of population initialization is calculated by O(3·N·D), where *N* is the population size and *D* is the dimensionality of the problem. Incorporating Latin Hypercube Sampling-based and opposition learning-based population generation adds an extra load to computer processors. Yet, its effectiveness in producing diverse members at the initial phase eases the convergence of the iterative process. The manipulation of candidate solutions through the MGO algorithm has the complexity of O(N·D). The proposed adaptive switch mechanism guiding the improved QDOPP algorithm variant has computational complexity O(2·N·D). If low and negligible complexities are eliminated, and only significant complexities are considered for the entire range of the iterations (*T*), then the overall complexity of the algorithm becomes $O\left(3\cdot N\cdot D\right)+O(3\cdot N\cdot D\cdot T)$. However, it is worth mentioning that the proposed search scheme is applied to the base MGO with a random probability of 0.5, which transforms the total complexity into its current and more reliable form of $O\left(3\cdot N\cdot D\right)+O(2\cdot N\cdot D\cdot T)$.

## 5. Simulation Results and Discussion

This section compares the proposed HCQDOPP-MGO algorithm with different Opposition-Based Learning-enhanced versions of MGO and newly emerging state-of-the-art metaheuristic algorithms. Detailed information about the configurational settings of tunable algorithm parameters will be provided for a fair comparison between the metaheuristic optimizers. Convergence analysis and box plot simulations based on statistical results are comparatively investigated to verify the superiority of the HCQDOPP-MGO. For simplicity in titling the proposed method, the abbreviated form of HCQDOPP is considered.

### 5.1. Benchmark Problems

This research study considers 28 test problems used in CEC 2013 competitions and 22 benchmark functions employed in the CEC 2014 competitions. Benchmark problems used in CEC competitions can be favorable alternatives to standard test functions, as they offer a greater diversity of problem types, including separable, non-separable, rotated, shifted, and noisy functions. In addition, they are abstract formulation models that can incorporate the complexities of real-world problems, rendering them a promising scenario for comprehending the capabilities of the developed algorithms. The CEC 2013 problems comprise five unimodal, fifteen multimodal, and eight composite test problems, each with a defined search space of [−100, 100]^D^. Unimodal test problems have only one critical optimum point within the defined search landscape, which is both a global and local point. In contrast, multimodal problems have multiple local minima or critical points within the search region. Considered CEC2014 test problems consist of 22 functions with distinctive characteristics; the first three are unimodal test problems with rotated versions of standard test problems; the fourth to sixteenth problems are shifted and rotated versions of standard test problems; and the remaining problems from seventeen to twenty-two are hybrid functions composed of integration of two or more standard test problems. Hybrid functions can be practical tools for benchmarking the maintained balance between exploration and exploitation mechanisms since each is constructed by combining multimodal and unimodal test problems. Variables are split into subgroups, and each decision variable set belonging to the respective subset is assigned to a different base function. This is an exemplary case that models the complexities of real-world problems, where different subsets have the potential to influence the objective in various ways. Like the previous case, the defined search domain is restricted to [−100, 100]^D^ for each problem in the CEC2014 test suite. Descriptions of the employed CEC 2013 and CEC 2014 benchmark functions are, respectively, given in Table 2 and Table 3.

### 5.2. Parameter Settings of the Comparative Algorithms

In recent years, numerous metaheuristic algorithms have emerged, each with varying search capacities due to their distinct governing search equations, which are enhanced by various complementary mathematical models incorporating unpredictable randomness. To perform a comparative study between the proposed improved MGO algorithm, this study considers nine relatively recently emerged metaheuristic optimizers, including the Manta Ray Foraging Optimization Algorithm (MANTA) [52], Runge–Kutta Optimizer (RUNGE) [53], African Vultures Optimization Algorithm (AVOA) [54], Gannet Optimization Algorithm (GANNET) [55], Electric-Eel Optimizer (EEL) [56], Equilibrium Optimizer (EQUIL) [57], Gradient-Based Optimizer (GRAD) [58], Reptile Search Algorithm (REPTILE) [59], and Coati Optimization Algorithm (COATI) [60]. A comparative study also extends to the different variants of Opposition-Based Learning algorithms embedded into standard MGO algorithms, which include standard Opposition-Based Learning (OBL) [25], Quasi Opposition-Based Learning (QOBL) [61], Quasi-Dynamic Opposition-based Learning (QDOPP), Super Opposition-Based Learning (SOBL) [62], Centroid Opposition-Based Learning (COBL) [63], and Multi-Individual Opposition-Based Learning (MIOBL) [64] search procedures. Table 4 provides the default parameter settings of the state-of-the-art algorithms considered for performance evaluations. Opposition-Based Learning variants lack tunable algorithm parameters, which are prolific tools for enhancing the diversity of the base algorithm’s population.

### 5.3. Comparison of the Statistical Results

**Figure 5:**
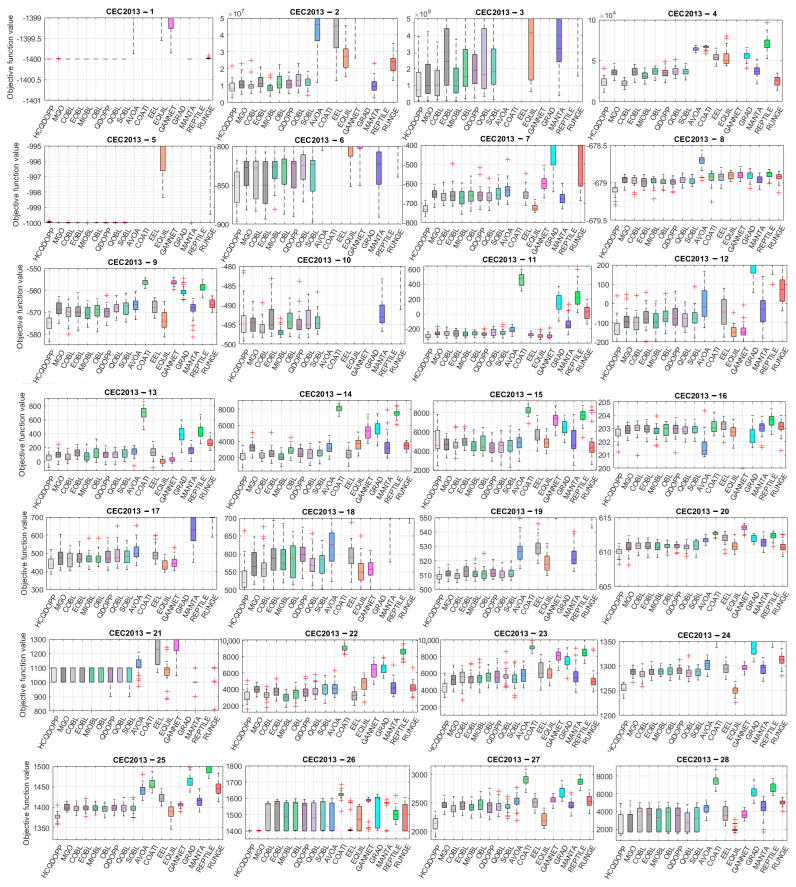
Statistical comparison of the competing algorithms for CEC 2013 problems.

**Figure 6:**
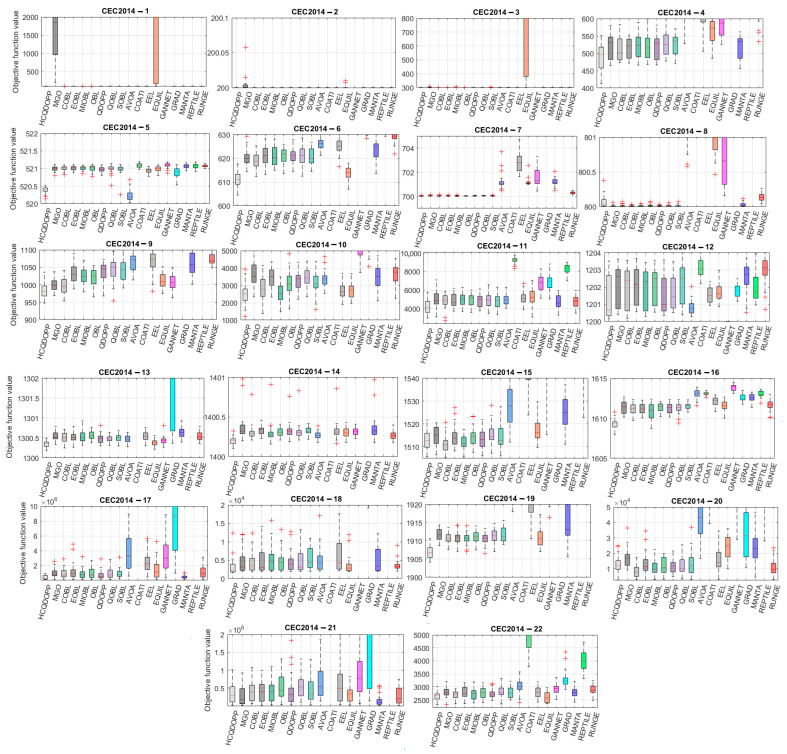
Box plot results of the compared algorithms for CEC 2014 test problems.

This section evaluates the convergence analysis of the proposed HCQDOPP against cutting-edge metaheuristic algorithms from the literature, using benchmark problems from the CEC 2013 and CEC 2014 competitions. When considering the CEC2013 and CEC2014 benchmark problems, 10,000 function evaluations are performed for each comparative algorithm, with 30 independent runs, due to the relatively higher complexities and nonlinearities of these test instances. The scalability of the compared algorithms will also be analyzed in the upcoming section, which serves as another reasonable indicator of the superiority of a metaheuristic optimizer. Figure 5 shows the box plot representation of the statistical results from the 30D benchmark problems utilized in the CEC 2013 competitions. In general, it is observed that different OBL-improved variant-enhanced MGO algorithms provide significantly better predictions compared to those obtained from state-of-the-art optimizers. The success of the MGO in obtaining accurate estimations is a direct outcome of its compelling exploration through navigation over multimodal landscapes and the precise refinement of promising regions detected during the diversification phase, which are significantly superior to those of the competing optimizers used in the comparison in Figure 5. HCQDOPP obtains the most accurate estimations in 20 out of 28 30D test problems and becomes the dominant algorithm among the methods compared. Nevertheless, the proposed method fails to yield accurate predictions for the following test instances: CEC2013-04 (Rotated Discus function), CEC2013-05 (Different Powers function), CEC2013-06 (Rotated Rosenbrock function), CEC2013-10 (Rotated Griewank function), CEC2013-16 (Rotated Katsuura function), and CEC2013-23 (Composition Function 3). Total collapse during the convergence process is observed for CEC2013-03 (Rotated Bent Cigar function) for all compared optimizers. This test problem features an elongated, stretched landscape, exhibiting extreme difficulties, particularly in higher dimensions, where even slight deviations make significant differences in objective function values. Additionally, the rotated landscape employs an algorithm for a general search space, which slows down the overall convergence speed. Nearly all algorithms fail to capture the correct trends as they converge to the optimal solution. Similar convergence tendencies are observed for CEC2013-02 (Rotated High-Conditioned Elliptic Function) and CEC2013-04 (Rotated Discus Function). These two unimodal functions exhibit functional characteristics of non-separability via rotation, making it nearly impossible to reach the optimum solution by treating each variable independently and forcing the algorithm to probe the coupled search space. Furthermore, narrow valleys and sharp ridges generated by highly curved spaces require a delicate balance between global exploration and local refinement to reach fertile regions where the most optimal solutions reside. All algorithms, including the proposed method and its variants, struggle to overcome the challenges of these functions and accurately estimate these test problems. Despite its significant failure in unimodal test problems, HCQDOPP provides relatively higher precision for predicting composite functions from CEC2013-21 to CEC2013-28, obtained after only 10,000 function evaluations for each problem, which is very low compared to the various applications in the literature. Figure 6 compares the statistical results for the contender algorithms on the 30D CEC2014 test problems in a box-plot representation. HCQDOPP and the remaining algorithms find the known global optimal solutions of CEC2014-01, CEC2014-02, CEC2014-03, CEC2014-07, and CEC2014-08 at least once after 10,000 function evaluations, which is a notable achievement for any metaheuristic in solving such complex unimodal and multimodal test problems. Considering the mean results, HCQDOPP performs best in 13 out of 22 test cases and outperforms the remaining algorithms in terms of solution robustness. Although HCQDOPP shows poor performance relative to the compared algorithms in terms of mean results for the CEC2014-05, CEC2014-08, and CEC2014-16 test problems, the results obtained are close to the known global optimum answers. Large deviations are observed between the best and worst predictions for hybrid functions of CEC2014-17, CEC2014-18, and CEC2014-21 test problems for the compared methods. It appears that algorithms struggle to adapt to these cases, particularly in accommodating diverse landscapes within a single function while maintaining a proper shift mechanism for diversification and intensification phases. HCQDOPP is more effective than the most competitive method among the 30D CEC2014 test problems when considering the best results.

### 5.4. Scalability Analysis and Statistical Test Results

**Figure 7:**
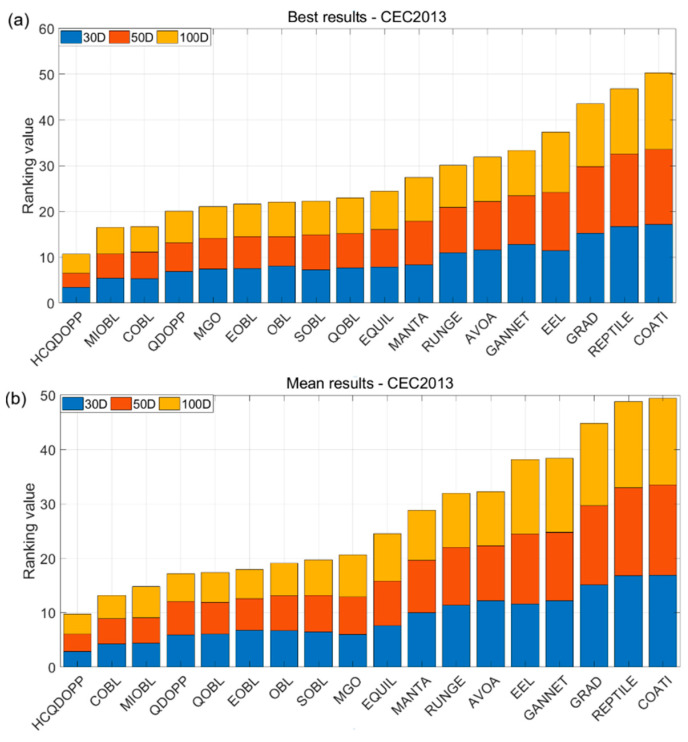
Ranking of the performance of the contender algorithms concerning the (**a**) best and (**b**) mean results.

**Figure 8:**
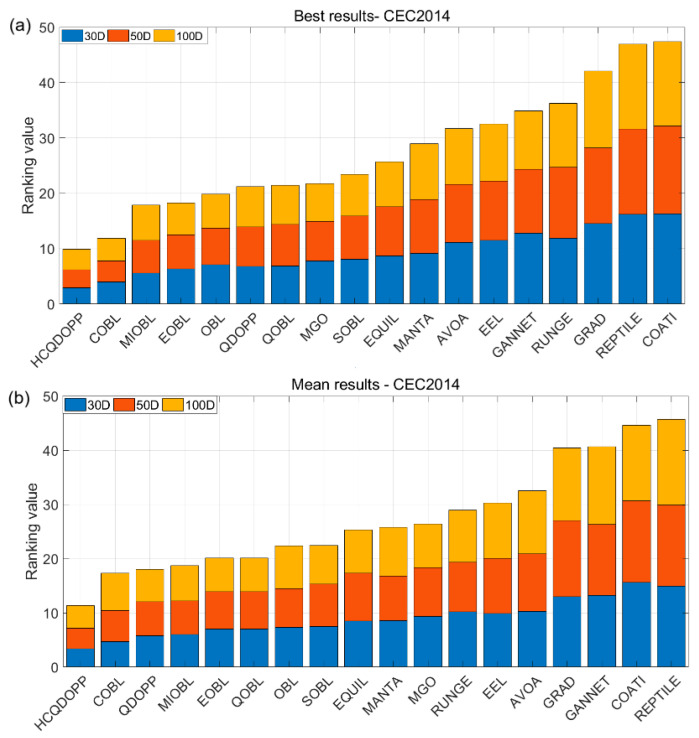
Overall optimization performance of the compared algorithms for different problem dimensionalities with respect to the (**a**) best and (**b**) mean results.

This section evaluates the performance variations of the proposed method and other remaining optimizers in the case of increasing problem dimensionalities of the employed test functions. The ranking analysis is performed to detect significant differences between multiple datasets, which are statistical results of the contender algorithms obtained for 30D, 50D, and 100D variants of the CEC2013 and CEC2014 benchmark problems, as well as standard test problems with 1000D, 2000D, and 3000D versions. Figure 7 shows the corresponding rankings of the compared algorithms for varying problem dimensionalities of the CEC 2013 test problems concerning both mean and best mean results. HCQDOPP ranks first among the competitive algorithms for two scenarios and proved superior over the remaining methods for CEC 2013 problems. The second-best algorithm, considering the best predictions for 30D, 50D, and 100D problems, is MIOBL. However, when mean results are considered, COBL algorithms rank second, while MIOBL ranks third as the best optimizer. GRAD, REPTILE, and COATI optimizers are the worst predictors for CEC 2013 problems. Figure 8 visualizes the comparison of the ranking results of the proposed method with those of other contestant algorithms for the CEC 2014 benchmark problems. HCQDOPP continues to dominate in terms of mean and best results for the 30D, 50D, and 100D benchmark functions, while the second-best algorithm is COBL. REPTILE and COATI algorithms again yield the most deviated predictions among the employed algorithms.

The Wilcoxon signed-rank test [65], an effective and practical statistical tool for assessing the significance of differences between two datasets, is used to verify the applicability of the HCQDOPP algorithm. Table 5 presents the statistical results of the Wilcoxon signed-rank test at significance levels of 0.05 and 0.1, comparing HCQDOPP with other algorithms across various test problems. The respective signs “+”, “=”, and “−” correspondingly indicate that the proposed HCQDOPP is better than, comparable to, and worse than its designated competitor. The *p*-values obtained for HCQDOPP across various test instances are below 0.05, confirming the statistical significance of HCQDOPP across all test functions employed in the performance comparison. In conclusion, HCQDOPP is the most competitive algorithm for various benchmark problems, significantly surpassing the contestant algorithms in terms of cumulative ranking performance and Wilcoxon test results.

### 5.5. Performance Assessment on CEC2006 Constrained Test Problems

In this section, the optimization capability of the proposed method will be verified on twelve test functions retrieved from the suite of CEC 2006-constrained problems. These are widely recognized benchmark suites, frequently utilized to evaluate the optimization performance of algorithms on constrained test cases. These problems can be considered practical test beds to assess the algorithm’s ability to handle various functional challenges, such as coping with nonlinear equality and inequality constraints, balancing feasibility and optimality, and overcoming the inherent multimodality of the test problems to avoid premature convergence. Table 6 presents the functional characteristics of the considered test functions for evaluating the performance of the compared algorithms. In Table 6, D is the problem dimensionality, LI is the number of linear inequality constraints, NI is the number of nonlinear inequality constraints, LE is the number of linear equality constraints, NE is the number of nonlinear equality constraints, and *f_op_*_t_(x) stands for the best-known optimal solution of the defined test problem.

Table 7 reports the statistical results of different state-of-the-art literature optimizers including MANTA, Marine Predators Algorithm (MARINE) [66], MGO, AVOA, Dandelion Optimizer (DANDEL) [67], EQUIL, Honey Badger Optimizer (HBADGER) [68], Kepler Optimization (KEPLER) [69], Mantis Search (MANTIS) [70], RUNGE, Slime Mould Optimizer (SLIME) [71], and Walrus Optimizer (WALRUS) [72], which are obtained after 10,000 function evaluations of 30 independent algorithm run.

HCQDOPP achieves the best predictions for G04, G09, G10, G13, G14, and G19 problems, while obtaining the second-best estimations for G01, G03, G07, and G18 test problems in terms of mean results, and it becomes the superior algorithm with a respective average ranking point of 1.666. The MANTIS algorithm is the second-best method, with a corresponding ranking value of 2.833. WALRUS is the worst predictor among them, with a ranking point of 10.971. It is observed that the MGO, KEPLER, and WALRUS optimizers fail to find any feasible solution in 30 consecutive runs. The G03 test problem challenges the applied algorithm due to its complex mathematical structure. Although this problem has only one active constraint, ensuring precise equality constraint satisfaction can be troublesome, as many optimization algorithms struggle to comply with this requirement. Minor deviations in decision variables may render the optimal solution infeasible. In addition, combining a nonlinear problem objective and nonlinear equality constraints can result in a highly non-convex feasible optimal region, making it challenging to explore. Algorithms with unbalanced feasibility and optimality struggle to find optimal solutions in feasible areas and may collapse at certain stages of the iteration.

## 6. A Complex Real-World Optimization Case: A Shell and Tube Heat Exchanger Design Operated with Nanofluids

**Figure 9:**
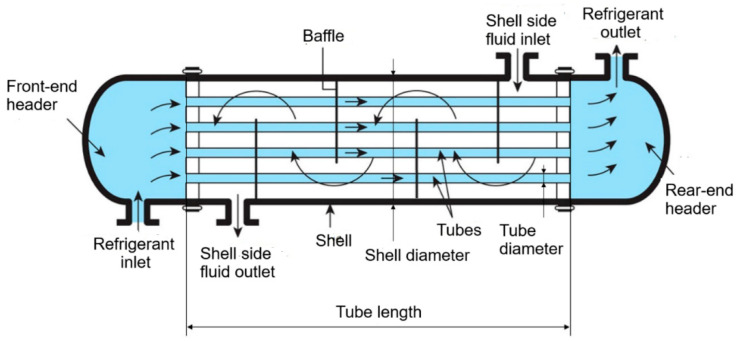
Schematic view of a shell and tube heat exchanger.

This section is primarily associated with the thermo-economic design of a shell-and-tube heat exchanger operating with different nanofluids on the tube side. Designing any heat exchanger is challenging and time-consuming, as the entire thermal design process requires complying with numerous restrictive thermal and structural constraints that must meet the expected requirements of end-users and working practitioners. The defined optimization problem involves highly nonlinear design objectives that must satisfy several constraints to achieve optimal construction. In this case, the primary motivation behind using a nanofluid-based refrigerant rather than a standard in-tube fluid is to investigate the valuable effects of the nanofluid’s influence on the overall heat exchange rates between the shell and tube side streams. However, one critical case that should be thoroughly scrutinized is the detrimental effects of the suspended nanoparticles in the nanofluid. A designer should make a plausible trade-off between the improved heat transfer rates and increased pressure drop rates resulting from the inclusion of nanoparticle effects. To evaluate the concurrent influences of nanoparticles on heat transfer and pressure drop rates, a comparative study is conducted among different heat exchanger configurations to determine which configuration yields the best thermo-economic performance under the specified heat load. The following section describes the heat transfer model employed in this study, which also considers the contribution of nanoparticle effects, providing end-users and practitioners with insights into the nanoparticle-based configuration that yields the most efficient design in total energy cost. Figure 9 illustrates a schematic of a shell-and-tube heat exchanger, labeling the essential components of the main construction.

### 6.1. Representative Heat Transfer Model

This section presents the primary heat exchanger equations used in the simulation. The total heat transfer rate is expressed using the *ε*-NTU approach as follows [73]

(39) $$Q=\varepsilon {\cdot C}_{min}\cdot \left(T_{h,i}-T_{c,i}\right)$$

where *C_min_* indicates the minimum heat capacity value; *T_h_*_,*i*_ and *T_c_*_,*i*_ are the hot and cold side inlet temperatures, respectively; and *ε* represents the effectiveness, which is the ratio between the actual heat transfer and the maximum heat transfer between two running streams [74] and can be formulated by

(40) $$\varepsilon =\frac{Q}{Q_{max}}$$

where *Q* is the current heat transfer rate and is only available if the inlet and outlet conditions of the running streams are known in advance or an iterative procedure is applied to obtain the actual heat transfer rate.

(41) $$Q=C_{hot}\left(T_{hot,i}-T_{hot,o}\right)=C_{cold}\left(T_{cold,o}-T_{cold,i}\right)$$

Alternatively, by employing ε–*NTU*, the actual heat exchange value can also be redefined by the following:

(42) $$Q=\varepsilon \cdot C_{min}\cdot \left(T_{hot,i}-T_{cold,i}\right)$$

For any heat exchanger, effectiveness is the direct function of the Number of Transfer Units (*NTU*s) and the ratio between the minimum and maximum heat capacity rates.

(43) $$\varepsilon =f\left(NTU,\frac{C_{min}}{C_{max}}\right)$$

where the heat capacity ratio is defined as

(44) $$C_{r}=\frac{C_{min}}{C_{max}}$$

The number of transfer units (*NTUs*) can be calculated as follows.

(45) $$NTU=\frac{U\cdot A_{o}}{C_{min}}$$

The total heat transfer area $A_{o}$ is calculated by

(46) $$A_{o}=N_{t}\pi d_{o}L_{t}$$

where *L_t_* represents tube length, and *N_t_* symbolizes the number of tubes, which can be obtained as follows [75].

(47) $$N_{t}=C{\left(\frac{D_{s}}{d_{o}}\right)}^{n_{1}}$$

Here, the coefficients *C* and *n*_1_ are model constants based on tube configuration and the number of tube passes [76].

The overall heat transfer coefficient is evaluated as follows [74].

(48) $$U_{o}=\frac{1}{\frac{1}{h_{s}}+R_{sf}+\frac{d_{o}ln\left(\frac{d_{o}}{d_{i}}\right)}{2k_{w}}+R_{tf}\frac{d_{o}}{d_{i}}+\frac{1}{h_{t}}\frac{d_{o}}{d_{i}}}$$

(49) $$d_{i}=d_{o}-2\cdot \delta$$

*R_sf_* and *R_tf_* are the shell and tube side fouling factors; *δ* is the tube thickness; *h_s_* and *h_t_* indicate the shell and tube side heat transfer coefficient. To evaluate the *U_o_* value of Equation (48), the *h_s_* and *h_t_* should be calculated beforehand. A convective heat transfer coefficient of nanofluid is used as the working fluid for the tube side. Additionally, the Dittus–Boelter equation [76] calculates the tube-side heat transfer coefficient–*h_t_*

(50) $${Nu}_{nf}=\frac{h_{t}d_{i}}{k}=0.023\cdot {Re}^{0.8}\cdot {Pr}^{0.4}$$

where the dimensionless Reynolds *Re* and Prandtl *Pr* numbers can be obtained as follows:

(51) $$Pr=\frac{\mu \cdot C_{p}}{k}$$

(52) $$Re=\frac{\dot{m}\cdot d_{i}}{A_{o}\cdot \mu }$$

The thermophysical properties of the nanofluids are evaluated based on the mean bulk temperature of the nanofluids. Additionally, the viscosity of water is 0.000759 kg/ms. The main thermophysical properties of the materials, which are the thermal conductivity, specific heat, and density that generate *Pr* in Equation (51) and the viscosity that is used to compute *Re* defined in Equation (52), are calculated in the presence of nanofluid using correlations obtained from literature.

The thermal conductivity of nanofluids can be computed by using the following correlation [77]

(53) $$k_{nf}=\frac{k_{f}\left(k_{p}+2k_{f}+2\left(k_{p}-k_{f}\right)\varphi \right)}{k_{p}+2k_{f}-\left(k_{p}-k_{f}\right)\varphi }$$

where *φ* represents the volume concentration, and the nanofluid-specific heat is determined as follows [78]:

(54) $$c_{p,nf}=\frac{\rho_{f}c_{p,f}\left(1-\varphi \right)+\rho_{p}c_{p,p}\varphi }{\rho_{nf}}$$

Here, *ρ_nf_* stands for the density of nanofluids, which can be evaluated using the following correlation [78]:

(55) $$\rho_{nf}=\varphi \rho_{p}+\left(1-\varphi \right)\rho_{f}$$

The viscosity of nanofluid can be found as follows [78]:

(56) $$\mu_{nf}=\mu_{f}\left(1+2.5\varphi \right)$$

This work considers six nanofluids—Al_2_O_3_, CuO, TiO_2_, Cu, SiO_2_, and Boehmite—as working fluids on the tube side. The thermophysical properties of these considered nanofluids are given in Table 8. Then, the effectiveness of the counter-current flow heat exchanger, which is also considered for the main configuration for two running streams in this study, is computed by:

(57) $$\varepsilon =\frac{1-exp\left(-NTU\left(1-C_{r}\right)\right)}{1-C_{r}\cdot exp\left(-NTU\left(1-C_{r}\right)\right)}$$

Water is utilized as a working fluid. Shell-side investigation is not straightforward because it involves complex and intricate flow characteristics. The Bell–Delaware approach, as described by Kakac et al. [76], is utilized to evaluate the convective heat transfer coefficient. This method considers five different leakages and bypass streams when assessing the convective heat transfer coefficient and pressure drop.

The essential equation for computing the convective heat transfer coefficient is as follows.

(58) $$h_{s}=h_{id}{\cdot Y}_{c}\cdot Y_{l}\cdot Y_{b}{\cdot Y}_{s}\cdot Y_{r}$$

Here, *Y_c_* corrects the irregularities related to baffle configuration; *Y_l_* represents the baffle leakage effects; *Y_b_* is the parameter responsible for the bypass effect; and *Y_s_* accounts for the change in baffle spacing at the inlet and outlet sections compared to the central baffle spacing. *Y_r_* is the factor that can be applied when *Re_s_* is less than 100. Table 9 illustrates these representative correction factor formulations and the parameters involved in these equations. *h_id_* stands for the ideal heat transfer coefficient, considering the assumption that the fluid is a pure crossflow along the tube bank; *h_id_* can be calculated as follows [76].

(59) $$h_{id}=j_{1}c_{ps}\left(\frac{\dot{m_{s}}}{A_{o,cr}}\right){\left(\frac{k_{s}}{c_{ps}\mu_{s}}\right)}^{2/3}{\left(\frac{\mu_{s}}{\mu_{s,w}}\right)}^{0.14}$$

where *A_o_*_,*cr*_ is the crossflow area. The parameter *j*_1_ represents the Colburn *j*-factor for an ideal tube bank, which can be obtained as follows:

(60) $$j_{1}=a_{1}{\left(\frac{1.33}{P_{t}/d_{o}}\right)}^{\frac{a_{3}}{1+0.14{\left({Re}_{s}\right)}^{a_{4}}}}{\left({Re}_{s}\right)}^{a_{2}}$$

Here, *α*_1_, *α*_2_, *α*_3_, and *α*_4_ symbolize the model parameters whose exact calculation procedure can be obtained from Kakac et al. [76].

In the STHE configuration, the shell side pressure drop consists of three different regions: the pressure drop in the entrance crossflow zone, Δ*p_cr_*, the pressure drop in the window section, Δ*p_w_*, and the pressure drop in the inlet and outlet part, Δ*p_e_*. The total pressure drop over the shell side is estimated using the Bell–Delaware approach, which combines these three factors in a single equation below.

(61) $$\Delta P_{s}=\Delta P_{cr}+\Delta P_{w}+\Delta P_{e}=\left(N_{b}-1\right)\Delta P_{b,id}\zeta_{b}\zeta_{l}+N_{b}\Delta P_{w,id}\zeta_{l}+2\Delta P_{b,id}\left(1+\frac{N_{r,cw}}{N_{r,cc}}\right)\zeta_{b}\zeta_{s}$$

Here, *N_b_* denotes the number of baffles; *N_r_*_,*cc*_ represents the number of tube rows taking place in one crossflow part, *N_r_,_cw_* is the number of tube rows occurring in each window. Parameters $\zeta_{l}$,$\;\zeta_{b}$, and $\zeta_{s}$ are correction factors. Δ*P_b,id_* symbolizes the pressure drop in an equivalent tube bank in the window part and can be found as follows.

(62) $${\Delta P}_{b,id}=\frac{4\cdot f_{id}\cdot G_{s}^{2}\cdot N_{r,cc}}{2\cdot \rho_{s}}{\left(\frac{\mu_{sw}}{\mu_{s}}\right)}^{0.25}$$

where *f_id_* represents the friction factor and is calculated based on the following equations.

(63) $$f_{id}=b_{1}{\left(\frac{1.33}{P_{t}/d_{o}}\right)}^{\frac{b_{3}}{1+0.14{\left({Re}_{s}\right)}^{b_{4}}}}{\left({Re}_{s}\right)}^{b_{2}}$$

In Equation (63), the numerical values of the model constraints *b*_1_, *b*_2_, *b*_3_, and *b*_4_ can be retrieved from Kakac et al. [76]. In Equation (64), Δ*P_w,id_* represents the pressure drop regarding one window section and is computed as follows.

(64) $$\Delta P_{w,id}=\frac{\left(2+0.6N_{r,cw}\right)\dot{m_{s}^{2}}}{2\cdot \rho_{s}\cdot A_{s}\cdot A_{o,w}}$$

The tube-side pressure drop can be calculated by the equation below [74]

(65) $$\Delta p_{t}=\frac{\dot{m_{t}}}{2\cdot \rho_{nf}\cdot A_{o,t}^{2}}\left[\frac{4\cdot f\cdot L}{d_{i}}+\left(1-\sigma^{2}+K_{c}\right)-\left(1-\sigma^{2}-K_{e}\right)\right]n_{p}$$

where *K_e_* and *K_c_* are sudden expansion and sudden contraction coefficients, *σ* is a function of the contraction ratio whose calculation method is described in Shah and Sekulic [74], *n_p_* is the number of tube passes, and *f* is the fanning friction factor, computed by the formulation

(66) $$f=0.046{Re}_{t}^{-0.2}$$

After defining the respective mathematical models for heat transfer and pressure rates resulting from the circulation of the running streams across the heat exchanger tubes, the primary design objective should be clearly defined to facilitate the optimization process. Minimizing the total heat exchanger cost is the primary design objective that the proposed optimization method, HCQDOPP, aims to maintain in this study. Defined objective function *C_tot_* is the total cost of the heat exchanger, which is comprised of the capital investment cost *C_c_*_,*inv*_ and operational costs *C_op_*.

(67) $$C_{tot}=C_{c,inv}+C_{op}$$

where the capital investment cost *C_c_*_,*inv*_ is computed by employing the Hall equation, which is defined by [79]

(68) $$C_{c,inv}=8000+259.2A_{o}^{0.93}$$

The following equation can calculate the total discounted cost expenditures related to the overall pumping power.

(69) $$C_{op}=\sum_{j=1}^{ny}\frac{C_{o}}{{\left(1+i\right)}^{j}}$$

(70) $$C_{o}=P_{pump}\cdot C_{EC}\cdot H$$

(71) $$P_{pump}=\frac{1}{\eta }\left(\frac{\dot{m_{t}}}{\rho_{t}}\Delta P_{t}+\frac{\dot{m_{s}}}{\rho_{s}}\Delta P_{s}\right)$$

where *C_o_* stands for the annual operating cost of the heat exchanger to be optimized; *i* is the annual interest rate; *ny* refers to the number of active years in which the heat exchanger is operated; *P_pump_* represents the consumed pumping power; *C_EC_* is the total energy cost in €/kWh; and *H* is the annual operational hours of the operating heat exchanger.

### 6.2. Optimization Results and Related Discussion

The HCQDOPP algorithm has been utilized to minimize the total cost of shell-and-tube heat exchangers. In this context, the Bell–Delaware method is adopted to solve the design problem, as this approach is considered one of the most effective design strategies available in the literature for handling the structural error that causes alternating flow currents and leakages.

The examined problem of a shell-and-tube heat exchanger has a heat load of 391.3 kW, as determined by the imposed heat transfer rate. Oil serves as the heat transfer fluid in the shell side, while the nanofluid circulates through the smooth tubes. Here, in this case, the main aim in examining this complex design process is to investigate how different in-tube flowing nanofluids influence the overall thermo-economic performance of a shell and tube heat exchanger and to gain valuable insights on the total structural and investment cost increases resulting from the inclusion of the suspended nanoparticles in the running refrigerant water stream in the tubes. A comprehensive comparative study discusses the thermo-economic efficiency of six different configurations, including Al_2_O_3_, CuO, TiO_2_, Cu, SiO_2_, and Boehmite nanofluids flowing on the tube side. Each different configuration is evaluated separately in terms of total cost considerations. The best-performing nanofluid configuration among the six available alternatives is decided based on the minimum cost expenditure. Another critical point to be scrutinized in this case is the feasibility of leveraging the merits of nanoparticles to reduce overall costs. It is of utmost importance to maintain a balance between the increased pumping power rates induced by the nanoparticle’s influence, caused by increased friction factor values, and the amount of increased heat transfer rates between the shell and tube side streams, which also result from the nanoparticle effects. To achieve the most feasible configuration, a successful designer should find a plausible trade-off between these contradictory and decisive design considerations. Additionally, definitive design constraints are established for the shell and tube side pressure drop rates to prevent excessive electrical costs and expenditures associated with the compressor’s operation. In this context, the pressure drop rate at the shell side must not exceed 25 kPa and should not exceed 6.0 kPa at the tube side. Another design constraint is imposed on the total heat exchange area, which should not exceed 50 m^2^. The last design constraint is subject to the pitch ratio, which must not violate the limit value of 1.25 times the tube outside diameter. The operational conditions of the shell-and-tube heat exchanger for six different nanofluid-based configurations are reported in Table 10. The upper and lower search limits for the sixteen design parameters of the shell-and-tube heat exchanger are reported in Table 11. Thirteen decision parameters are continuous, while the remaining three are integers. Table 12 presents the optimal values of the decision parameters obtained by the proposed HCQDOPP algorithm for various heat exchanger configurations. The proposed HCQDOPP has performed 50 algorithm runs, each with 50,000 function evaluations, for each heat exchanger configuration whose optimal design parameters are reported in Table 12, along with the optimal values of decisive model parameters.

It is observed that the heat exchanger design with SiO_2_ + water nanofluid running in the tubes has the lowest total cost of € 21,116.82 compared to the other remaining configurations, which also include the preliminary design operated with pure water for in-tube flow. In terms of minimum total cost, the second-best heat exchanger design involves a configuration operating with Al_2_O_3_ + water nanofluid on the tube side. Among the six contestant heat exchanger configurations, the worst design alternative in terms of overall cost values is operated with water and Boehmite nanofluid, having a minimum fitness value of 24,915.59 €. This is only marginally better than the preliminary design, which operates with pure water, with a total cost value of € 25,231.71. When total discounted capital cost and operating cost rates are thoroughly examined, all six different design configurations have approximately similar capital investment cost rates, which are directly related to the total area of the heat exchanger. As is known, the total area of the heat exchanger is directly associated with the overall heat transfer coefficient values, which are also quite similar between the compared design configurations. Therefore, it can be concluded that the decisive factor influencing the total cost rates is the discounted operating cost values, which are a direct function of the pumping power (*P_pump_*) resulting from the pressure drop across the shell side and tube side flows. As shown in Table 12, the heat exchanger configuration operating with water + SiO_2_ yields the lowest shell side pressure rate (Δ*p_shell_*) of 11,961.01 Pa and a moderate tube side pressure rate (Δ*p_t_*) of 5522.524 Pa, resulting in a total discounted operating cost rate of € 4815.064. As mentioned earlier, the total heat exchange surface significantly impacts the investment cost rate of a shell-and-tube heat exchanger. The configuration operated with SiO_2_ + water has the overall investment cost rate of 16,301.76 €, which is better than those obtained from the configurations running with pure water (16,298.73 €), and water + Al_2_O_3_ (16,525.932 €), water + CuO (16,946.78 €), water + TiO_2_ (16,488.13 €), and water + Cu (16,615.21 €) nanofluids.

One can also observe a significant decrease in total cost values (16.3%) when water + SiO_2_ nanofluid is utilized in the tube side. This primarily results from the substantial decline in total discounted operating cost rates (46.1%) compared to the preliminary design, which is operated with pure water in tubes. This decrease is attributed to the reductions in shell-side (50.3%) and tube-side (4.5%) pressure drop values. The SiO_2_ nanoparticle ratio of 4.69% in the water + SiO_2_ nanofluid significantly contributes to the tube-side pressure rates, which are entailed by the increased friction between the nanofluid and adjacent tube walls. The total heat exchanger surface of the configuration running with water and SiO_2_ is negligibly higher than that of the preliminary design, resulting in an insignificant increase in investment cost values.

**Figure 10:**
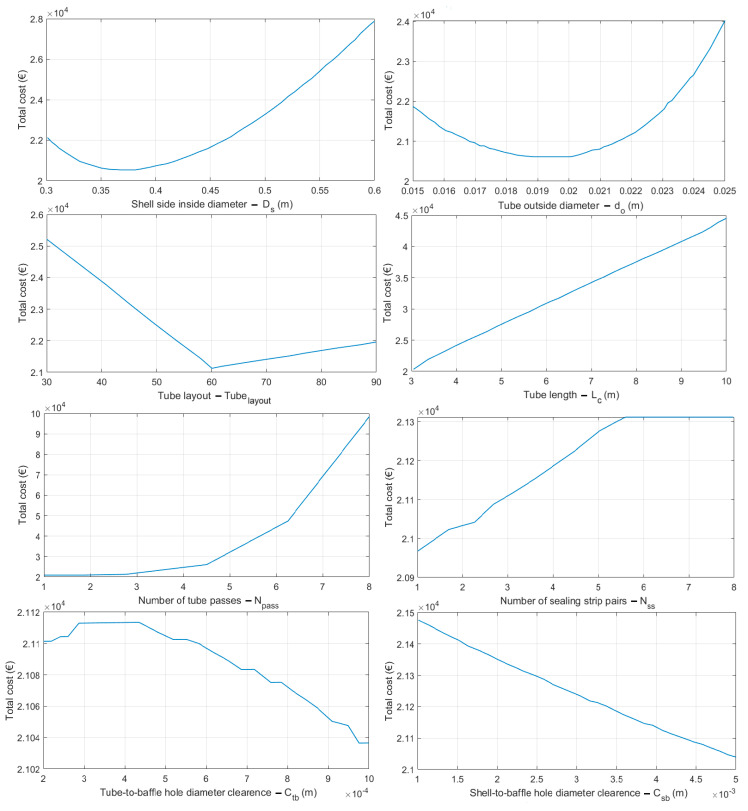
Influences of design variables on total cost rates.

**Figure 11:**
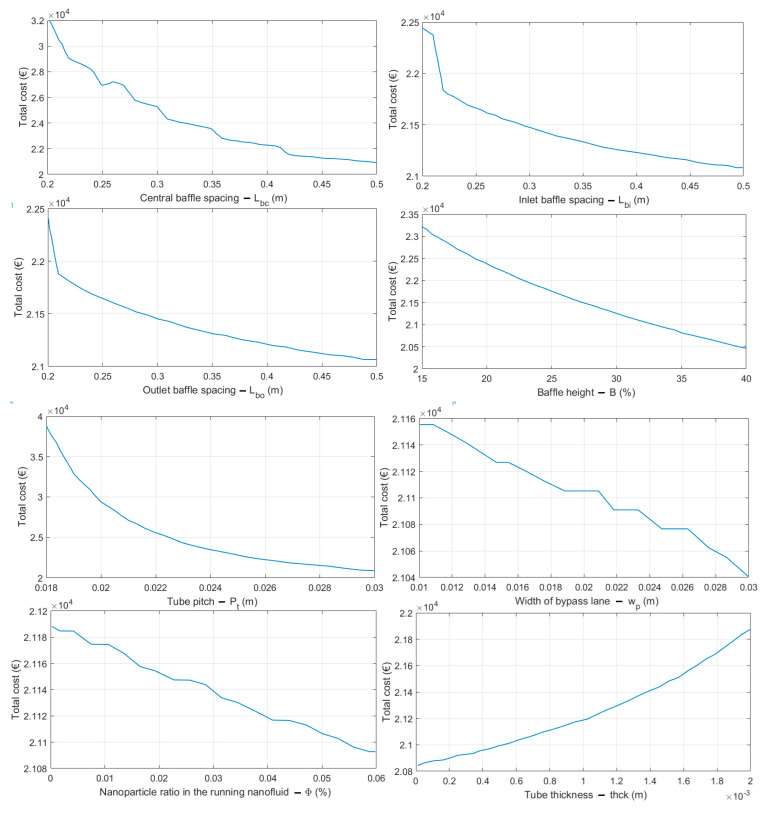
Variations of heat exchanger cost values with increasing values of design parameters.

Figure 10 and Figure 11 illustrate the parametric analysis of the sixteen decision parameters, whose numerical behaviors are examined as the design variables vary from lower to upper bounds. In these figures, an increase in the shell side and tube outside diameters leads to a decline in total case rates. However, considerable increases are observed as these design parameter values are increased to their upper limits. The total cost rate is the maximum when the tube layout is 30° and takes its minimum value when the tube layout is 60°. The increase in tube length entails an expected increase in total cost rates due to the increase in the total heat exchange area. An increase in the number of tubes results in a considerable increase in total cost values due to the increased pressure drop rates on both the tube and shell sides, as well as the total heat exchange area. It is also observed that variations in the number of sealing strip pairs, tube-to-baffle hole diameter clearance, and shell-to-baffle hole diameter clearance, within their allowed bounds, have a negligible influence on total cost rates. Relatively massive declines are observed in total cost rates when the design variable of central baffle spacing varies from its lower to upper search limit. However, changes in the objective function of total cost rates are comparatively minor when outlet baffle spacing and inlet baffle spacing design variables vary from lower to upper search limits. Total cost rates are reduced when the height of the baffles in the heat exchanger increases. It is worth mentioning that tube pitch, which refers to the distance between adjacent tubes in a tube bundle, is a crucial factor in shell-and-tube heat exchangers, as significant declines are observed when tube pitch values are increased to their maximum allowable limit. It is also seen that the bypass lane width has a negligible impact on total cost rates. Changes in tube thickness have a negligible effect on total cost rates. One crucial issue that is extensively scrutinized is the effect of nanoparticles on heat transfer and pressure drop rates. As discussed in the preceding paragraphs, integrating nanoparticles into the refrigerant fluid has a variable impact on these two essential design parameters, decreasing total cost rates as the volumetric nanoparticle ratio increases from lower to upper bounds. End users, researchers, and designers should remember that an increase in suspended nanoparticles in the refrigerant stream enhances the heat transfer coefficient values on the tube side. However, it also entails increased pressure drop rates, negatively influencing the tube-side pressure drop rates. When nanoparticle effects are considered, a plausible balance should be maintained in pressure drop and heat transfer coefficient rates to obtain the minimum total cost value.

## 7. Conclusive Remarks

This study proposes two novel variants of Quasi-Dynamic Oppositional-based Learning methods to be integrated into the newly emerging metaheuristic optimizer, the Mountain Gazelle Optimizer, to enhance its overall optimization performance. At the initial phase, where population initialization commences, a combination of Latin Hypercube sampling, empowered by Logistic chaotic sequences, and basic principles of the Opposition-based Learning mechanism is considered to generate highly diverse, evolvable candidate solutions before proceeding to the consecutive iterations. To further enhance the overall solution quality in the gazelle population, chaotic numbers generated from various chaotic maps have been separately embedded into the corresponding search equations to assess their impact on the total solution quality. Twenty-one chaos-induced Mountain Gazelle Optimizers have solved forty-eight standard optimization benchmark problems, comprising unimodal and multimodal test functions. The best-performing chaotic variant among them has been decided based on its success rate over the employed test problems. The second phase of the algorithm development procedure involves enhancing the overall solution accuracy of the best chaotic variant of the Mountain Gazelle Optimizer, utilizing a novel mutation scheme that leverages the concurrent contributive influences of two different improved versions of Quasi-Dynamic Opposition Learning algorithms, which is administered by a novel adaptive switch mechanism, responsible for selecting which variants should be employed at the current iteration, decided by taking into account the previous successes of the two competing algorithms. The proposed mutation equation has significantly increased solution diversity in the iteratively evolving population, dramatically improving the solution qualities of applied test problems, which include the most renowned benchmark functions used in CEC 2006, CEC 2013, and CEC 2014 competitions.

Finally, the enhanced algorithm comprehensively investigates and optimizes a full-fledged shell-and-tube exchanger with various nanofluids. The optimization problem is inherently a mixed-integer design case, involving thirteen continuous and three integer decision variables, significantly increasing the problem’s complexity. As a significant contribution to the existing literature, the comparative performance of different heat exchanger configurations operating with various nanofluids has been investigated, and the proposed algorithm has been utilized for structural and topological optimization. The following remarkable conclusions can be drawn from this theoretical research study.

- When ranking points of the competitive chaotic Mountain Gazelle Optimization algorithm variants are averaged to a mean ranking point for each optimization problem, it is seen that integration of the chaotic numbers produced from the CH02 (Bernoulli map) yields the best predictive results of the employed forty-eight unimodal and multimodal test problems with different problem dimensionalities.
- The proposed intelligently guided manipulation scheme has significantly improved the solution diversity within the population, thanks to the unpredictable yet conducive features of the Opposition-Based Learning, Quasi-Dynamic Opposition-Based Learning, and Quasi Opposition-Based Learning methods, all three of which have complementary capabilities that can eliminate the algorithmic disadvantages of each method by the created synergy between them during the hybridization process. Numerical simulations demonstrate that shaping these three innovative learning schemes into a solid, structured form renders them so dexterous and prolific that the proposed search strategy has acquired the ability to explore unexplored regions of the search space without incurring excessive computational costs. Comprehensive evaluations of the proposed mutation scheme’s versatility suggest that it can enhance the overall optimization performance of any metaheuristic optimizer, thereby demonstrating the method’s efficiency on a global scale.
- It is also understood that Opposition-Based Learning has been proven effective in improving metaheuristic algorithms for optimizing complex structural design problems, such as finding the optimal configuration of a shell and tube heat exchanger or other challenging real-world design cases. The highly randomized characteristics of these improved methods make it effortless to obtain optimal solutions to complex design problems with high nonlinearity and binding, restrictive constraints.
- A detailed investigation of the influences of streaming nanofluids in the tubes of a shell-and-tube heat exchanger indicates that suspended nanoparticles in the refrigerant fluid can increase the tube-side heat transfer rates to some degree. However, it can also increase the tube-side pressure drop rates, which necessitates carefully weighing the optimum amount of nanoparticles in the nanofluid, as both the tube-side heat transfer coefficient and pressure drop rates directly affect the total cost of a heat exchanger.
- Among the six available design alternatives, a heat exchanger configuration operating with a water + SiO_2_ nanofluid on the tube side yields the minimum total cost rate, thanks to its superior thermophysical properties.
- As a reasonable future projection inspired by this research study, it would be beneficial for metaheuristic algorithm developers to focus on the mutation equations based on the integration of two or more oppositional learning search mechanism variants since they are capable of making abrupt movements in the search space to avoid the local optimum points encountered throughout the iterative process.

## Figures and Tables

**Table 1:** Description of the multimodal and unimodal test functions with varying search ranges.

Multimodal Function Name	Range	Unimodal Function Name	Range
*F*_1_—Ackley	[−32, 32] ^D^	*F*_25_—Sphere	[−5.12, 5.12] ^D^
*F*_2_—Griewank	[−600, 600] ^D^	*F*_26_—Rosenbrock	[−30.0, 30.0] ^D^
*F*_3_—Rastrigin	[−5.12, 5.12] ^D^	*F*_27_—Brown	[−1.0, 4.0] ^D^
*F*_4_—Zakharov	[−5.0, 10.0] ^D^	*F*_28_—Stretched V Sine Wave	[−10.0, 10.0] ^D^
*F_5_*—Alpine	[0, 10] ^D^	*F*_29_—Powell	[0.0, 10.0] ^D^
*F*_6_—Penalized1	[−50.0, 50.0] ^D^	*F*_30_—Sum of Different Powers	[−1.0, 1.0] ^D^
*F*_7_—Csendes	[−5.0, 5.0] ^D^	*F*_31_—Bent cigar	[−5.0, 5.0] ^D^
*F*_8_—Schaffer	[−100.0, 100.0]	*F*_32_—Discus	[−100.0, 100.0] ^D^
*F*_9_—Salomon	[−50.0, 50.0] ^D^	*F*_33_—Schwefel 2.20	[−100.0, 100.0] ^D^
*F*_10_—Inverted cosine mixture	[−10.0, 10.0] ^D^	*F*_34_—Schwefel 2.21	[−100.0, 100.0] ^D^
*F*_11_—Wavy	[−3.14, 3.14] ^D^	*F*_35_—Schwefel 2.23	[−10.0, 10.0] ^D^
*F*_12_—Xin She Yang1	[−5.0, 5.0] ^D^	*F*_36_—Schwefel 2.25	[0.0, 10.0] ^D^
*F*_13_—Xin She Yang4	[−6.28, 6.28] ^D^	*F*_37_—Dropwave	[−5.12, 5.12] ^D^
*F*_14_—Xin She Yang2	[−10.0, 10.0] ^D^	*F*_38_—Trid	[D^2^, D^2^] ^D^
*F*_15_—Pathological	[−10.0, 10.0] ^D^	*F*_39_—Generalized White & Holst	[−10.0, 10.0] ^D^
*F*_16_—Quintic	[−10.0, 10.0] ^D^	*F*_40_—BIGGSB1	[−10, 10] ^D^
*F*_17_—Levy	[−10.0, 10.0] ^D^	*F*_41_—Anescu01	[−2.0, 2.0] ^D^
*F*_18_—Qing	[−500.0, 500.0] ^D^	*F*_42_—Anescu02	[1.39, 4.0] ^D^
*F*_19_—Diagonal1	[−10.0, 10.0] ^D^	*F*_43_—Anescu03	[−4.0, 1.39] ^D^
*F*_20_—Hager	[−10.0, 10.0] ^D^	*F*_44_—Anescu04	[0.001, 2.0] ^D^
*F*_21_—Diagonal4	[−10.0, 10.0] ^D^	*F*_45_—Anescu06	[0.001, 2.0] ^D^
*F*_22_—Perturbed Quadratic Diagonal	[−10.0, 10.0] ^D^	*F*_46_—Anescu07	[−2.0, 2.0] ^D^
*F*_23_—SINE	[−10.0, 10.0] ^D^	*F*_47_—Schumer-Steiglitz 3	[−100.0, 100.0] ^D^
*F*_24_—Diagonal9	[−10.0, 10.0] ^D^	*F*_48_—Schumer-Steiglitz 2	[−100.0, 100.0] ^D^

D—Problem dimension.

**Table 2:** Summary of CEC 2013 test problems.

	No	Functions	Global Optimum–*f*(x)
	1	Sphere function	−1400
Unimodal	2	Rotated High Conditioned Elliptic Function	−1300
Functions	3	Rotated Bent Cigar Function	−1200
	4	Rotated Discus Function	−1100
	5	Different Powers Function	−1000
	6	Rotated Rosenbrock’s Function	−900
	7	Rotated Schaffers F7 Function	−800
	8	Rotated Ackley’s Function	−700
	9	Rotated Weierstrass Function	−600
	10	Rotated Griewank’s Function	−500
Basic	11	Rastrigin’s Function	−400
Multimodal	12	Rotated Rastrigin’s Function	−300
Functions	13	Non-Continuous Rotated Rastrigin’s Function	−200
	14	Schwefel’s Function	−100
	15	Rotated Schwefel’s Function	100
	16	Rotated Katsuura Function	200
	17	Lunacek–Bi-Rastrigin Function	300
	18	Rotated Lunacek–Bi-Rastrigin Function	400
	19	Expanded Griewank’s plus Rosenbrock’s Function	500
	20	Expanded Scaffer’s F6 Function	600
	21	Composition Function 1 (*n* = 5, Rotated)	700
	22	Composition Function 2 (*n* = 3, Unrotated)	800
Composite	23	Composition Function 3 (*n* = 3, Rotated)	900
Functions	24	Composition Function 4 (*n* = 3, Rotated)	1000
	25	Composition Function 5 (*n* = 3, Rotated)	1100
	26	Composition Function 6 (*n* = 5, Rotated)	1200
	27	Composition Function 7 (*n* = 5, Rotated)	1300
	28	Composition Function 8 (*n* = 5, Rotated)	1400

Search Range: [−100, 100]^D^ D: problem dimension.

**Table 3:** Descriptions of the employed CEC 2014 test problems.

	No	Functions	Global Optimum–*f*(x)
	1	Rotated High Conditioned Elliptic Function	100
Unimodal	2	Rotated Bent Cigar Function	200
Functions	3	Rotated Discus Function	300
	4	Shifted and Rotated Rosenbrock’s Function	400
	5	Shifted and Rotated Ackley’s Function	500
	6	Shifted and Rotated Weierstrass Function	600
	7	Shifted and Rotated Griewank’s Function	700
	8	Shifted Rastrigin’s Function	800
	9	Shifted and Rotated Rastrigin’s Function	900
	10	Shifted Schwefel’s Function	1000
Basic	11	Shifted and Rotated Schwefel’s Function	1100
Multimodal	12	Shifted and Rotated Katsuura Function	1200
Functions	13	Shifted and Rotated HappyCat Function	1300
	14	Shifted and Rotated HGBat Function	1400
	15	Shifted and Rotated Expanded Griewank’s plus Rosenbrock’s Function	1500
	16	Shifted and Rotated Expanded Scaffer’s F6 Function	1600
	17	Hybrid Function 1 (*n* = 3)	1700
	18	Hybrid Function 2 (*n* = 3)	1800
Hybrid	19	Hybrid Function 3 (*n* = 4)	1900
Functions	20	Hybrid Function 4 (*n* = 4)	2000
	21	Hybrid Function 5 (*n* = 5)	2100
	22	Hybrid Function 6 (*n* = 5)	2200

Search Range: [−100, 100]^D^ D: problem dimension.

**Table 4:** Default parameter settings of the comparative algorithms.

Algorithms	Parameters
AVOA	p_1_ = 0.6, p_2_ = 0.4, p_3_ = 0.6, α = 0.8, β = 0.2, γ = 2.5
GANNET	M = 2.5, Velocity = 1.5
REPTILE	α = 0.1, β = 0.1
EQUIL	a_1_ = 2.0, a_2_ = 1.0, GP = 0.5
COATI	No tunable algorithm parameters
EEL	No tunable algorithm parameters
GRAD	No tunable algorithm parameters
MANTA	No tunable algorithm parameters
RUNGE	No tunable algorithm parameters
MGO	No tunable algorithm parameters

**Table 5:** Statistical results for CEC2013 and CEC2014 (30D, 50D, 100D) benchmark problems.

HCQDOPP vs.	Wilcoxon Signed-Rank Test					
	+	−	=	*p*-Value	α = 0.05	α = 0.1
COATI	141	6	3	<0.05	Yes	Yes
REPTILE	140	5	5	<0.05	Yes	Yes
GANNET	139	7	4	<0.05	Yes	Yes
GRAD	141	6	3	<0.05	Yes	Yes
AVOA	136	8	6	<0.05	Yes	Yes
EEL	134	7	9	<0.05	Yes	Yes
MANTA	137	7	6	<0.05	Yes	Yes
EQUIL	132	10	8	<0.05	Yes	Yes
MGO	134	10	6	<0.05	Yes	Yes
OBL	133	9	8	<0.05	Yes	Yes
SOBL	135	9	6	<0.05	Yes	Yes
QOBL	131	10	9	<0.05	Yes	Yes
EOBL	127	13	10	<0.05	Yes	Yes
QDOPP	129	11	10	<0.05	Yes	Yes
MIOBL	130	10	10	<0.05	Yes	Yes
COBL	125	15	10	<0.05	Yes	Yes

**Table 6:** Functional characteristics of the employed CEC 2006 constrained test problems.

	Type	D	LI	NI	LE	NE	*f_opt_* (*x*)
G01	Quadratic	13	9	0	0	0	−15.000000
G02	Nonlinear	20	0	2	0	0	−0.8036191
G03	Polynomial	10	0	0	0	1	−1.0005001
G04	Quadratic	5	0	6	0	0	−30,665.538
G06	Cubic	2	0	2	0	0	−6961.8138
G07	Quadratic	10	3	5	0	0	24.306209
G09	Polynomial	7	0	4	0	0	680.63005
G10	Linear	8	3	3	0	0	7048.24802
G13	Nonlinear	5	0	0	0	3	0.05394151
G14	Nonlinear	10	0	0	3	0	−47.764888
G18	Quadratic	9	0	13	0	0	−0.8660254
G19	Nonlinear	15	0	5	0	0	32.6555929

**Table 7:** Comparison of the statistical results for CEC2006 constraint test problems.

		HCQDOPP	MANTA	MARINE	MGO	AVOA	DANDEL	EQUIL	HBADGER	KEPLER	MANTIS	RUNGE	SLIME	WALRUS
G01	Best	−1.500 × 10^1^	−1.499 × 10^1^	−1.499 × 10^1^	−1.500 × 10^1^	−1.275 × 10^1^	−1.299 × 10^1^	−1.483 × 10^1^	−1.425 × 10^1^	−1.492 × 10^1^	−1.497 × 10^1^	−1.446 × 10^1^	−1.499 × 10^1^	−1.483 × 10^1^
	Mean	−1.500 × 10^1^	−1.033 × 10^1^	−1.496 × 10^1^	−1.500 × 10^1^	−9.764 × 10^0^	−8.686 × 10^0^	−1.402 × 10^1^	−1.151 × 10^1^	−1.243 × 10^1^	−1.492 × 10^1^	−1.252 × 10^1^	−1.153 × 10^1^	−9.454 × 10^0^
	Std	4.632 × 10^−5^	3.039 × 10^0^	3.752 × 10^−2^	0	1.462 × 10^0^	1.585 × 10^0^	8.060 × 10^−1^	1.173 × 10^0^	2.444 × 10^0^	4.832 × 10^−2^	9.763 × 10^−1^	2.026 × 10^0^	2.264 × 10^0^
	rank	2	10	3	1	11	13	5	9	7	4	6	8	12
G02	Best	−7.842 × 10^−1^	−7.682 × 10^−1^	−7.878 × 10^−1^	−7.241 × 10^−1^	−7.818 × 10^−1^	−7.508 × 10^−1^	−7.977 × 10^−1^	−7.807 × 10^−1^	−7.444 × 10^−1^	−7.906 × 10^−1^	−7.741 × 10^−1^	−6.698 × 10^−1^	−6.502 × 10^−1^
	Mean	−7.312 × 10^−1^	−6.645 × 10^−1^	−7.354 × 10^−1^	−5.521 × 10^−1^	−6.456 × 10^−1^	−5.971 × 10^−1^	−7.473 × 10^−1^	−6.836 × 10^−1^	−6.204 × 10^−1^	−7.261 × 10^−1^	−6.823 × 10^−1^	−5.632 × 10^−1^	−4.851 × 10^−1^
	Std	3.097 × 10^−2^	8.249 × 10^−2^	3.505 × 10^−2^	8.650 × 10^−2^	8.144 × 10^−2^	6.564 × 10^−2^	3.525 × 10^−2^	4.411 × 10^−2^	6.350 × 10^−2^	4.941 × 10^−2^	5.737 × 10^−2^	3.911 × 10^−2^	4.286 × 10^−2^
	Rank	3	7	2	12	8	10	1	5	9	4	6	11	13
G03	Best	−9.058 × 10^−1^	−7.680 × 10^−1^	−1.908 × 10^−1^	N/A	−1.462 × 10^−1^	−8.390 × 10^−2^	−2.971 × 10^−1^	−1.440 × 10^−1^	N/A	−5.955 × 10^−1^	−3.105 × 10^−1^	−1.000 × 10^0^	N/A
	Mean	−2.878 × 10^−1^	−9.094 × 10^−2^	−3.652 × 10^−2^	N/A	−1.405 × 10^−2^	−3.818 × 10^−3^	−5.114 × 10^−2^	−2.046 × 10^−2^	N/A	−7.851 × 10^−2^	−5.196 × 10^−2^	−1.000 × 10^0^	N/A
	Std	2.393 × 10^−1^	1.310 × 10^−1^	5.024 × 10^−2^	N/A	3.257 × 10^−2^	1.449 × 10^−2^	6.136 × 10^−2^	3.444 × 10^−2^	N/A	1.121 × 10^−1^	7.927 × 10^−2^	0	N/A
	rank	2	3	7	13	9	10	6	8	13	4	5	1	13
G04	Best	−3.066 × 10^4^	−3.066 × 10^4^	−3.066 × 10^4^	−3.066 × 10^4^	−3.062 × 10^4^	−3.066 × 10^4^	−3.066 × 10^4^	−3.065 × 10^4^	−3.066 × 10^4^	−3.066 × 10^4^	−3.066 × 10^4^	−3.066 × 10^4^	−3.066 × 10^4^
	Mean	−3.066 × 10^4^	−3.066 × 10^4^	−3.066 × 10^4^	−3.065 × 10^4^	−3.035 × 10^4^	−3.065 × 10^4^	−3.066 × 10^4^	−3.058 × 10^4^	−3.066 × 10^4^	−3.066 × 10^4^	−3.059 × 10^4^	−3.066 × 10^4^	−3.065 × 10^4^
	Std	5.825 × 10^−3^	1.360 × 10^0^	1.732 × 10^−1^	3.078 × 10^1^	2.175 × 10^2^	4.046 × 10^1^	4.193 × 10^1^	4.560 × 10^1^	1.143 × 10^−1^	5.724 × 10^−1^	5.833 × 10^1^	1.589 × 10^0^	4.858 × 10^1^
	rank	1	5	3	8	13	7	10	12	4	2	11	6	9
G06	Best	−6.961 × 10^3^	−6.961 × 10^3^	−6.961 × 10^3^	−6.961 × 10^3^	−6.955 × 10^3^	−6.960 × 10^3^	−6.961 × 10^3^	−6.961 × 10^3^	−6.961 × 10^3^	−6.961 × 10^3^	−6.961 × 10^3^	−6.961 × 10^3^	−6.953 × 10^3^
	Mean	−6.961 × 10^3^	−6.942 × 10^3^	−6.961 × 10^3^	−6.961 × 10^3^	−6.771 × 10^3^	−6.951 × 10^3^	−6.943 × 10^3^	−6.956 × 10^3^	−6.903 × 10^3^	−6.961 × 10^3^	−6.960 × 10^3^	−6.957 × 10^3^	−6.877 × 10^3^
	Std	7.055 × 10^−3^	9.623 × 10^0^	2.407 × 10^−1^	9.400 × 10^−7^	7.553 × 10^2^	6.473 × 10^0^	1.349 × 10^1^	3.912 × 10^0^	2.768 × 10^2^	1.206 × 10^−2^	1.391 × 10^0^	4.309 × 10^0^	6.939 × 10^1^
	rank	3	9	4	2	13	8	10	7	11	1	5	6	12
G07	Best	2.445 × 10^1^	2.437 × 10^1^	2.435 × 10^1^	2.443 × 10^1^	2.595 × 10^1^	2.512 × 10^1^	2.462 × 10^1^	2.638 × 10^1^	2.452 × 10^1^	2.441 × 10^1^	2.550 × 10^1^	2.530 × 10^1^	2.625 × 10^1^
	Mean	2.475 × 10^1^	2.612 × 10^1^	2.469 × 10^1^	2.712 × 10^1^	4.238 × 10^1^	2.827 × 10^1^	2.716 × 10^1^	3.488 × 10^1^	2.501 × 10^1^	2.499 × 10^1^	3.236 × 10^1^	2.801 × 10^1^	3.133 × 10^1^
	Std	1.984 × 10^−1^	1.540 × 10^0^	2.216 × 10^−1^	2.048 × 10^0^	3.044 × 10^1^	3.011 × 10^0^	3.549 × 10^0^	8.601 × 10^0^	3.970 × 10^−1^	4.310 × 10^−1^	8.339 × 10^0^	2.519 × 10^0^	3.945 × 10^0^
	rank	2	5	1	6	13	9	7	12	4	3	11	8	10
G09	Best	6.806 × 10^2^	6.806 × 10^2^	6.806 × 10^2^	6.808 × 10^2^	6.819 × 10^2^	6.806 × 10^2^	6.806 × 10^2^	6.808 × 10^2^	6.806 × 10^2^	6.806 × 10^2^	6.806 × 10^2^	6.814 × 10^2^	6.813 × 10^2^
	Mean	6.806 × 10^2^	6.807 × 10^2^	6.806 × 10^2^	6.827 × 10^2^	6.899 × 10^2^	6.814 × 10^2^	6.808 × 10^2^	6.838 × 10^2^	6.806 × 10^2^	6.806 × 10^2^	6.834 × 10^2^	6.852 × 10^2^	6.844 × 10^2^
	Std	4.337 × 10^−3^	6.221 × 10^−2^	2.991 × 10^−2^	1.519 × 10^0^	9.938 × 10^0^	5.173 × 10^−1^	1.765 × 10^−1^	4.592 × 10^0^	2.086 × 10^−2^	2.182 × 10^−2^	1.688 × 10^0^	4.879 × 10^0^	2.155 × 10^0^
	rank	1	5	3	8	13	7	6	10	4	2	9	12	11
G10	Best	7.105 × 10^3^	7.145 × 10^3^	7.066 × 10^3^	7.261 × 10^3^	7.619 × 10^3^	7.503 × 10^3^	7.257 × 10^3^	7.535 × 10^3^	7.178 × 10^3^	7.096 × 10^3^	7.557 × 10^3^	7.676 × 10^3^	7.648 × 10^3^
	Mean	7.211 × 10^3^	7.907 × 10^3^	7.341 × 10^3^	8.137 × 10^3^	9.327 × 10^3^	8.786 × 10^3^	7.901 × 10^3^	8.290 × 10^3^	7.468 × 10^3^	7.389 × 10^3^	8.359 × 10^3^	8.933 × 10^3^	8.838 × 10^3^
	Std	8.199 × 10^1^	6.408 × 10^2^	1.976 × 10^2^	5.553 × 10^2^	1.191 × 10^3^	1.106 × 10^3^	3.619 × 10^2^	7.257 × 10^2^	2.188 × 10^2^	1.509 × 10^2^	5.715 × 10^2^	6.497 × 10^2^	7.369 × 10^2^
	rank	1	6	2	7	13	10	5	8	4	3	9	12	11
G13	Best	1.297 × 10^−1^	7.531 × 10^−2^	6.086 × 10^−2^	7.356 × 10^−2^	4.242 × 10^−1^	1.826 × 10^−1^	8.835 × 10^−2^	1.652 × 10^−1^	N/A	5.446 × 10^−2^	7.527 × 10^−2^	7.556 × 10^−1^	4.431 × 10^−1^
	Mean	3.414 × 10^−1^	5.663 × 10^−1^	2.786 × 10^−1^	8.042 × 10^−1^	7.657 × 10^−1^	8.090 × 10^−1^	7.819 × 10^−1^	7.304 × 10^−1^	N/A	2.497 × 10^−1^	6.734 × 10^−1^	9.619 × 10^−1^	8.094 × 10^−1^
	Std	1.833 × 10^−1^	2.847 × 10^−1^	1.581 × 10^−1^	2.621 × 10^−1^	2.081 × 10^−1^	2.554 × 10^−1^	2.616 × 10^−1^	2.867 × 10^−1^	N/A	1.766 × 10^−1^	2.939 × 10^−1^	8.488 × 10^−2^	2.038 × 10^−1^
	rank	1	4	2	9	7	10	8	6	13	1	5	12	11
G14	Best	−4.763 × 10^1^	−4.728 × 10^1^	−4.709 × 10^1^	−4.608 × 10^1^	−4.686 × 10^1^	−4.707 × 10^1^	−4.703 × 10^1^	−4.673 × 10^1^	−4.727 × 10^1^	−4.752 × 10^1^	−4.759 × 10^1^	N/A	−4.653 × 10^1^
	Mean	−4.697 × 10^1^	−4.576 × 10^1^	−4.630 × 10^1^	−4.482 × 10^1^	−4.486 × 10^1^	−4.365 × 10^1^	−4.511 × 10^1^	−4.462 × 10^1^	−4.666 × 10^1^	−4.676 × 10^1^	−4.424 × 10^1^	N/A	−4.438 × 10^1^
	Std	7.152 × 10^−1^	8.714 × 10^−1^	4.795 × 10^−1^	7.260 × 10^−1^	1.008 × 10^0^	1.758 × 10^0^	1.148 × 10^0^	1.147 × 10^0^	5.842 × 10^−1^	5.433 × 10^−1^	1.441 × 10^0^	N/A	1.122 × 10^0^
	rank	1	5	4	8	7	12	6	9	3	2	11	13	10
G18	Best	−8.626 × 10^−1^	−8.660 × 10^−1^	−8.660 × 10^−1^	−8.656 × 10^−1^	−8.617 × 10^−1^	N/A	−8.657 × 10^−1^	−7.704 × 10^−1^	−8.637 × 10^−1^	−8.657 × 10^−1^	−8.610 × 10^−1^	−8.642 × 10^−1^	−8.526 × 10^−1^
	Mean	−8.389 × 10^−1^	−7.115 × 10^−1^	−8.590 × 10^−1^	−8.107 × 10^−1^	−5.609 × 10^−1^	N/A	−7.020 × 10^−1^	−5.391 × 10^−1^	−7.964 × 10^−1^	−8.308 × 10^−1^	−6.052 × 10^−1^	−8.208 × 10^−1^	−6.413 × 10^−1^
	Std	2.238 × 10^−2^	1.427 × 10^−1^	8.768 × 10^−3^	8.711 × 10^−2^	1.199 × 10^−1^	N/A	1.419 × 10^−1^	1.099 × 10^−1^	9.135 × 10^−2^	6.708 × 10^−2^	1.232 × 10^−1^	8.417 × 10^−2^	1.606 × 10^−1^
	rank	2	7	1	5	11	13	8	12	6	3	10	4	9
G19	Best	3.640 × 10^1^	3.973 × 10^1^	4.031 × 10^1^	3.684 × 10^1^	4.595 × 10^1^	N/A	4.833 × 10^1^	3.963 × 10^1^	3.730 × 10^1^	4.166 × 10^1^	4.414 × 10^1^	3.521 × 10^1^	3.989 × 10^1^
	Mean	4.166 × 10^1^	5.776 × 10^1^	4.793 × 10^1^	5.816 × 10^1^	1.064 × 10^2^	N/A	6.579 × 10^1^	5.079 × 10^1^	4.626 × 10^1^	5.384 × 10^1^	7.811 × 10^1^	5.397 × 10^1^	7.276 × 10^1^
	Std	4.308 × 10^0^	1.112 × 10^1^	4.350 × 10^0^	1.629 × 10^1^	5.790 × 10^1^	N/A	1.203 × 10^1^	9.063 × 10^0^	5.127 × 10^0^	7.024 × 10^0^	1.819 × 10^1^	1.350 × 10^1^	2.083 × 10^1^
	rank	1	7	3	8	12	13	9	4	2	5	11	6	10
Aver.rank		1.666	6.083	2.916	7.250	10.833	10.167	6.750	8.500	6.666	2.833	8.25	8.25	10.971
Rankings		1	4	3	7	12	11	6	10	5	2	8	9	13

N/A indicates the associated algorithm cannot find any feasible solution in any of the consecutive runs.

**Table 8:** Thermophysical properties of suspended nanoparticles and water as a working base fluid [78].

Base Fluid				Nanoparticles			
Components	Water	Al_2_O_3_	CuO	TiO_2_	Cu	SiO_2_	Boehmite
*ρ* (kg/m^3^)	995	3970	6000	4250	8933	2220	3050
*C_p_* (J/kg.K)	4178	765	551	686	385	745	618.8
*k* (W/mK)	0.619	40	33	8.9	400	1.4	30

**Table 9:** Formulations of the correction factors employed in calculating Equation (58) of shell side heat transfer c, as follows.

Formula	Parameters Employed in the Equation
$Y_{c}=0.55+0.72F_{c}$	The value of *F_c_* can be found in Shah and Sekulic [74].
$Y_{l}=0.44\left(1-r_{s}\right)+\left[1-0.44\left(1-r_{s}\right)\right]exp(-2.2r_{m})$	$r_{s}=\frac{A_{o,sb}}{A_{o,sb}+A_{o,tb}}$$r_{lm}=\frac{A_{o,sb}+A_{o,tb}}{A_{o,cr}}$ Here, *A_o_*_,*sb*_, *A_o_*_,*tb*_, *A_o_*_,*cr*_ are given in Shah and Sekulic [74].
$Y_{b}=\left\{\begin{array}{c} 1\;\;\;\;\;\;\;\;\;\;\;\;\;\;\;\;\;\;\;\;\;\;\;\;\;\;for\;\;N_{ss}^{+}\geq 0.5\\ exp\left(-C\cdot r_{b}\cdot \left(1-{\left(2\cdot N_{ss}^{+}\right)}^{1/3}\right)\right)\;for\;\;N_{ss}^{+}<0.5 \end{array}\right.$	$r_{b}=\frac{A_{o,bp}}{A_{o,cr}}$ , $N_{ss}^{+}=\frac{N_{ss}}{N_{r,cc}}$ , $C=\left\{\begin{array}{c} 1.35\;\;\;for\;\;{Re}_{s}\leq 100\\ 1.25\;\;\;for\;\;{Re}_{s}>100 \end{array}\right.$ Explicit formulations of *N_ss_* and *N_r_*_,*cc*_ are given in Shah and Sekulic [74]
$Y_{s}=\frac{N_{b}-1+{\left(L_{i}^{+}\right)}^{\left(1-n\right)}+{\left(L_{o}^{+}\right)}^{\left(1-n\right)}}{N_{b}-1+{\;L}_{i}^{+}+{\;L}_{o}^{+}}$	$L_{i}^{+}=\frac{L_{bi}}{L_{bc}}$ , $L_{o}^{+}=\frac{L_{bo}}{L_{bc}}$ , $n=\left\{\begin{array}{c} 0.6\;\;\;for\;\;turbulent\;flow\\ 0.33\;\;for\;\;laminar\;\;flow \end{array}\right.$ *L_bi_*, *L_bo_*, and *L_bc_* are, respectively, baffle spacing at the inlet, outlet, and center
$Y_{r}=\left\{\begin{array}{c} 1\;\;\;\;\;\;\;\;\;\;\;\;for\;\;{Re}_{s}\geq 100\\ {\left(10/N_{r,c}\right)}^{0.18}\;\;for\;\;{Re}_{s}<100 \end{array}\right.$	$N_{r,c}=N_{r,cc}+N_{r,cw}$ , where *N_r_*_,*cc*_ and *N_r_*_,*cw*_ are calculated by the formulations given in Shah and Sekulic [74].

**Table 10:** Operational conditions of different heat exchanger configurations.

	Shell Side			Tube Side			
Process Fluids	Oil	Al_2_O_3_ + H_2_O	CuO + H_2_O	TiO_2_ + H_2_O	Cu + H_2_O	SiO_2_ + H_2_O	Boehmite + H_2_O
Flow rate (kg/s)	36.3	5.1	5.1	5.1	5.1	5.1	5.1
Inlet Temp. (°C)	65.6	32.2	32.2	32.2	32.2	32.2	32.2
Outlet Temp. (°C)	60.4	52.5	52.3	53.2	53.7	52.3	50.8
Density (kg/m^3^)	849	1080.88	1076.56	1114.83	1151.12	1052.49	997.34
Heat Capacity (J/kg.K)	2094	3816.14	3848.59	3687.92	3599.11	3838.18	4165.62
Viscosity (Pa.s)	0.0646	0.00081	0.00079	0.000829	0.000796	0.000848	0.000761
Thermal Conductivity (W/m.K)	0.14	0.67164	0.648068	0.676573	0.656078	0.645164	0.620988

**Table 11:** Upper and lower search bounds for the considered design parameters.

Parameter	Lower Bound	Upper Bound
Shell-side inside diameter—*D_s_* (m)	0.3	0.6
Tube-side outside diameter—*d_o_* (m)	0.012	0.025
Tube length—*L* (m)	3	10
Tube pitch—*p_t_* (m)	0.015	0.03
Central baffle spacing—*L_bc_* (m)	0.2	0.5
Inlet baffle spacing—*L_bi_* (m)	0.2	0.5
Outlet baffle spacing—*L_bo_* (m)	0.2	0.5
Baffle spacing (%)	15	40
Width of bypass lane—*w_p_* (m)	0.01	0.03
Tube-to-baffle hole diametral clearance—*δ_tb_* (m)	0.0001	0.001
Shell-to-baffle diametral clearance—*δ_sb_* (m)	0.001	0.005
Tube thickness—*thck* (m)	0.0002	0.002
The nanoparticle ratio—*φ*_v_ (%)	0	0.6
Number of tube passes—*N_pass_*	1 2 4 6 8	
Number of sealing strip pairs—*N_ss_*	1 2 4 8	
Tube layout—*T_layout_* (°)	30 45 90	

**Table 12:** Optimal values of the considered design parameters for various heat exchanger configurations.

	Water	Al_2_O_3_	CuO	TiO_2_	Cu	SiO_2_	Boehmite
Optimization Variables							
Shell-side inside diameter—*D_s_* (m)	0.458	0.456	0.451	0.495	0.433	0.429	0.457
Tube-side outside diameter—*d_o_* (mm)	22.9	17.5	16.8	20.4	17.2	16.3	22.3
Tube layout—*T_layout_* (°)	45	45	45	45	45	45	45
Number of tube passes—*N_pass_*	2	2	2	2	2	2	2
Tube length—*L* (m)	4.59	3.12	3.22	3.01	3.52	3.24	4.41
Tube pitch—*p_t_* (mm)	28.7	29.7	29.3	29.7	27.8	29.1	28.8
Central baffle spacing—*L_bc_* (m)	0.493	0.448	0.484	0.459	0.427	0.469	0.491
Inlet baffle spacing—*L_bi_* (m)	0.398	0.335	0.469	0.425	0.446	0.463	0.399
Outlet baffle spacing—*L_bo_* (m)	0.387	0.438	0.497	0.491	0.361	0.459	0.392
Baffle spacing (%)	41.242	30.932	33.372	26.832	39.324	31.632	39.873
Width of bypass lane—*w_p_* (mm)	14.9	23.8	13.7	19.3	27.6	17.4	15.2
Tube-to-baffle hole diametral clearance—*δ_tb_* (mm)	0.354	0.65	0.527	0.421	0.419	0.422	0.367
Shell-to-baffle diametral clearance—*δ_sb_* (mm)	3.562	3.762	3.289	3.361	2.772	4.183	3.601
Number of sealing strip pairs—*N_ss_*	8	2	1	2	8	2	8
Tube thickness—*thck* (mm)	1.2	1.2	1.6	1	0.7	1	1.2
The nanoparticle ratio—*φ*_v_ (%)	0.111	2.975	1.62	3.665	1.983	4.693	0.121
Model parameters							
Transverse tube pitch—*X_t_* (mm)	40.3	42.1	41.8	42.4	39.3	40.9	40.6
Longitudinal tube pitch—*X_l_* (mm)	20.3	21.5	20.7	21.3	19.7	20.5	20.4
Total number of tubes—*N*	149	277	297	246	256	271	151
Tube clearance—*Cl* (mm)	6.4	12.3	12.6	9.8	10.7	12.5	6.7
Shell side mass velocity—*G_s_* (kg/m^2^ s)	523.482	309.642	282.694	347.893	382.134	305.045	506.132
Shell side Reynolds number—*Re_s_*	278.333	117.932	101.987	153.245	142.832	107.99	249.783
Shell side heat transfer coefficient—*h_s_* (W/m^2^K)	531.892	519.983	481.563	542.981	512.782	531.697	526.891
Pressure drops in the central section—Δ*p_cr_* (Pa)	3601.374	2306.482	1824.698	3179.421	2581.232	2232.91	3533.792
Pressure drops in the window area—Δ*p_w_* (Pa)	10,193.742	5453.392	5192.784	5767.911	8041.273	5979.75	10,053.56
Pressure drops in inlet and outlet section—Δ*p_i-o_* (Pa)	9856.392	4471.744	3163.942	4796.472	5198.481	3748.34	9457.232
Total shell side pressure drop—Δ*p_shell_* (Pa)	24,083.974	12,231.42	10,181.84	13,740.14	15,817.8	11,961.0	23,042.74
Total number of baffles—*N_b_*	8	6	6	6	6	6	8
Tube-side Reynolds number—*Re_t_*	23,331.635	14,177.4	15,825.72	13,259.13	15,261.32	13,823.25	22,106.07
Tube-side heat transfer coefficient—*h_i_* (W/m^2^ K)	4421.732	4039.831	4960.753	3224.129	4009.93	4186.97	4384.231
Overall heat transfer coefficient—*U_o_* (W/m^2^ K)	409.572	400.535	379.753	405.223	398.184	410.464	408.932
Total heat transfer area—*A_o_* (m^2^)	45.113	46.484	48.421	46.78	47.532	45.127	45.001
Effectiveness (ε)	0.2675	0.2696	0.2641	0.2847	0.2905	0.2626	0.2441
Tube-side pressure drop—Δ*p_t_* (Pa)	5783.321	3899.932	6898.933	2181.42	4119.134	5522.524	5637.842
Annual operating cost—*C_o_* (€/year)	1423.848	760.231	713.123	806.071	958.283	783.63	1406.124
Total discounted operating cost—*C_oD_* (€)	8932.982	4672.832	4379.592	4947.941	5885.133	4815.06	8638.201
Capital investment cost—*C_i_* (€)	16,298.733	16,525.932	16,946.78	16,488.13	16,615.212	16,301.7	16,277.391
Total cost of heat exchanger—*C_tot_* (€)	25,231.71	21,198.76	21,326.37	21,436.07	22,500.345	21,116.13	24,915.591

## Data Availability

Source codes of the algorithms developed in this research study are available upon reasonable request.

## Supplementary Materials

Matlab codes of the chaotic Latin Hyper Cube sampling method described in Section 4.1, Hybrid Opposition-Based Learning Procedure provided in Algorithm 2, the proposed random number generator based on ISSM-RNG, explained in Equation (20), Adaptive Fitness Driven Perturbation algorithm explained in Algorithm 3, and Hybrid QDOPP-AFDP given in Algorithm 4 are publicly available and can be downloaded at: https://www.mdpi.com/article/10.3390/biomimetics10070454/s1.
